# Economic Evaluations of Speech and Language Therapy Interventions: A Scoping Review

**DOI:** 10.1111/1460-6984.70091

**Published:** 2025-07-12

**Authors:** James Hill, Amit Kulkarni, Deborah Moll, Lotte Meteyard, Louise Shelley, Grace Chugg, Gemma Jones, Valerio Benedetto, Catherine Harris, Alison Doherty, Joanna Harrison, Andrew Clegg

**Affiliations:** ^1^ Applied Health Research hub University of Central Lancashire and Methodological Innovation, Development Adaptation and Support (MIDAS) Theme, NIHR Applied Research Collaboration North‐West Coast (ARC NWC) Preston UK; ^2^ Royal College of Speech & Language Therapists London UK; ^3^ University of Central Lancashire Preston UK; ^4^ Oxford Health NHS Foundation Trust Oxford UK; ^5^ Department of Psychology and Clinical Language Sciences University of Reading Reading UK; ^6^ Humber Teaching NHS Foundation Trust Willerby UK; ^7^ Leeds Gender Identity Service Leeds UK

**Keywords:** Speech and Language Therapy, Economic Evaluation, Cost‐Effectiveness, Health Economics, Scoping Review

## Abstract

**Background:**

With constrained funding and increasing demand, the pressures on health and care services globally continue to grow. Given the challenges faced, it is imperative that services and interventions demonstrate cost‐effectiveness, as well as patient/service‐user and societal benefits, to maximize impact. Research has started to explore the cost‐effectiveness of speech and language therapy (SLT) interventions, but little is known about this body of evidence overall. Given the increasing demand for economic information by different decision‐makers, an overall understanding of the current evidence base is needed. Such an evaluation should map and assess the current evidence to identify key gaps, areas of strength, and recommendations for future economic evaluations of SLT.

**Aim:**

To identify the extent and type of economic evaluations assessing the cost (including cost‐effectiveness) of SLT interventions.

**Methods:**

A scoping review was conducted identifying studies across seven key databases from date of inception to 20 October 2023. Studies were included if they assessed any type of cost of an intervention which was primarily delivered (directly or indirectly) by a speech and language therapist. A narrative synthesis was undertaken and clustered around common populations of interest, including adults post‐stroke, adults with aphasia or dysarthria, adults with Parkinson's disease, adults with head and neck cancer, and children, young people, and adults with a range of conditions.

**Outcomes and Results:**

This scoping review identified 52 studies which have been published over three and a half decades. Recently, there has been a notable increase in the number of studies, specifically cost‐effectiveness and cost‐utility analyses; however, there were limited internal citations and a substantial variation in populations and interventions assessed by these studies. Economic evaluations were carried out alongside various effectiveness study designs, using different units of analysis, time horizons, and perspectives.

**Conclusion:**

Although a small number of studies have begun to explore the economic aspects of SLT, evidence in this area remains relatively scarce, highlighting the need to prioritize further economic evaluations. Given the substantial heterogeneity and limited interconnectivity within the existing evidence base, it is crucial to strategically organize and coordinate efforts to optimize future research. Future economic evaluations of SLT interventions should ideally run alongside randomized controlled trials and use decision‐analytic modelling techniques to strengthen the economic evidence base.

**WHAT THIS PAPER ADDS:**

*What is already known on the subject*

- As pressures grow on health and social care services, it is increasingly important that the care and support provided is shown to be both effective and cost‐effective, informing those who plan, provide, receive and pay for the services. Although some research has begun to examine the cost‐effectiveness of speech and language therapy (SLT) interventions, the overall extent and depth of this body of evidence remain unknown.

*What this study adds to the existing knowledge*

- This scoping review offers an overview of the maturity, depth, and extent of the evidence assessing the costs and cost‐effectiveness of interventions provided by SLT. Although the evidence base continues to grow, it appears to lack coherence in its development, necessitating a strategically planned approach to optimize and coordinate efforts to the benefit of the service and those using it.

*What are the potential or clinical implications of this work?*

- This scoping review has identified 52 studies which assessed the cost of SLT. The evidence from these studies can help us better understand the economic value of SLT. However, given the relative immaturity of the evidence base and lack of coordination across the findings, further research is required. This research should draw from the findings of this review and would ideally be part of a coherent, coordinated effort to develop a full understanding of the economic value of SLT across different clinical areas.

## 1 Introduction

In recent years, the costs associated with health and social care worldwide have been steadily increasing, presenting significant challenges to health and social care systems (Charlesworth et al. 2021; Lastuka et al. 2024; Muñoz et al. 2010; Nghiem and Connelly 2017; Watt et al. 2019). Despite a desire to reduce public spending in many countries across the world, healthcare expenditure has consistently risen in real terms over the past two decades (Harding and Pritchard 2016; Organisation for Economic Co‐operation and Development 2013; Watt et al. 2019; World Health Organization 2021). This has placed healthcare systems under substantial scrutiny (Department of Health and Social Care 2016; NHS 2016; World Health Organization 2021, 2024) and intensified the focus on efficiency. It in turn has led governments to prioritize the importance of implementing cost‐effective healthcare interventions (Canadian Agency for Drugs and Technologies in Health 2015; Haute Autorité de Santé 2020; Institute for Clinical Economic Review 2020; Institute for Quality Efficiency in Health Care 2020; National Institute for Health and Care Excellence 2022a).

Economic evaluations systematically combine data about the effects of an intervention with its associated costs to produce a single unit cost (e.g., Incremental Cost Effectiveness Ratio [ICER] (Anton and Lisa 2022), Cost per quality‐adjusted life year [QALY], Cost per disability‐adjusted life year [DALY] or Cost per life year gained [LYG]), enabling comparison between two or more alternative interventions (Anton and Lisa 2022). Assessing cost‐effectiveness enables a more comprehensive comparison of the effects of interventions, empowers decision‐makers to make optimal choices in maximizing service delivery within constrained resources, and facilitates efficient use of public funding (Cohen and Reynolds 2008; Cubi‐Molla et al. 2021; National Institute for Health and Care Excellence 2014).

Speech and language therapy (SLT) is fundamental in providing assessment, treatment, support and care as part of a range of services, including rehabilitation (National Institute for Clinical Excellence 2004; National Institute for Health and Care Excellence 2023b), social work (National Institute for Health and Care Excellence 2022b), mental health (National Institute for Health and Care Excellence 2016), criminal justice (Royal College of Speech and Language Therapists 2023) learning disabilities (Royal College of Speech and Language Therapists 2023) emergency care (Royal College of Speech and Language Therapists 2024) and education (Department for Children Schools and Families 2008; Public Health England 2020). As such, it is important that evidence on both the effectiveness and cost‐effectiveness of interventions in SLT is assessed.

The field of research on the economic value of SLT interventions is still evolving (de Sonneville‐Koedoot et al. 2017). Previous cost‐efffectiveness reviews in this area have resulted in very few economic evaluations being identified (Canadian Agency for Drugs and Technologies in Health 2015; Law et al. 2012; Tosh et al. 2017; Yang et al. 2023). These reviews have mainly focused on single approaches (e.g., telehealth for SLT (Canadian Agency for Drugs and Technologies in Health 2015)), parent‐implemented home therapy programmes (Tosh et al. 2017), interventions for a specific population (e.g., children with speech, language and communication needs (Law et al. 2012)), or approaches within a specific population (e.g., home‐based telerehabilitation interventions for dysphagia in patients with head and neck cancer (Yang et al. 2023)). Additionally, several systematic reviews within SLT have explicitly acknowledged the significant lack of research on cost‐effectiveness (Benfield et al. 2020; Brady et al. 2022; Brignell et al. 2021; Pennington et al. 2016). Given this identified paucity of evidence focusing specifically on cost‐effectiveness, a broader review is required to understand current evidence around any economic aspect of SLT. This scoping review aimed to identify the extent and type of studies (i.e., economic evaluations) assessing the cost (including cost‐effectiveness) of SLT interventions. The extent of studies includes an examination of the volume, timeline, geographical distribution, interconnectivity, scope and methods of studies evaluating the costs of SLT interventions.

## 2 Methods

To achieve the aim of the review, five sub‐questions have been proposed to explore the extent and type of studies (i.e., economic evaluations) assessing the cost (including cost‐effectiveness) of SLT interventions:

Q1: How has the volume and types of studies assessing the cost or cost‐effectiveness of SLT interventions evolved over time?
Q2: What is the geographical distribution of studies assessing the cost or cost‐effectiveness of SLT interventions?
Q3: What is the interconnectivity of studies assessing the cost or cost‐effectiveness of SLT interventions within the existing evidence base?
Q4: Which clinical populations have been the focus of studies assessing the cost or cost‐effectiveness of SLT interventions?
Q5: What are the key characteristics (e.g., study design, intervention type, evaluation methods, perspectives, and time horizons) of studies assessing the cost or cost‐effectiveness of SLT interventions?

### 2.1 Scoping Review Approach and Registration

This scoping review was pre‐registered on the Open Science Framework (https://osf.io/xvqp8). It followed methodological guidance provided by Peters et al. (2015) and Levac et al. (2010) and was reported in accordance with the PRISMA extension for scoping reviews (PRISMA‐ScR) guideline (see Supporting Information S1: Appendix 1 for checklist) (Tricco et al. 2018).

### 2.2 Search Strategy

The following databases were searched to identify relevant studies: Medline (Ovid), Embase (Ovid), CINAHL (EBSCOhost), PsycINFO (EBSCOhost), ERIC (EBSCOhost), Cochrane Library all databases (via Wiley), and the International HTA Database (Date of inception until 20/10/2023). The search strategy was formulated through collaboration between an information specialist, systematic review methodologists, health economists and SLT researchers and clinicians. It included subject headings and terms relating to SLT, which were combined with the Canadian Agency for Drugs and Technologies in Health (CADTH) Economic Evaluations and Models search filter to identify economic evaluation studies (Canadian Agency for Drugs and Technologies in Health 2015). The word ‘communication’ was not included, as such a search term was found to retrieve a high number of irrelevant papers. No date limits were applied to the searches. The searches were limited to English language due to the lack of translation facility access. An example of the full search strategy for Medline (Ovid) can be found in Supporting Information S1: Appendix 2.

To supplement the database searches, forward and backward citation searches were performed for all studies included at the full paper screening stage using the Web of Science citation index (on 06/02/2024). Results from the citation searches were deduplicated against records retrieved in the initial database search, and any papers which were not identified in the initial stage were screened for inclusion. Identification of duplicates was undertaken using EndNote initially and then Rayyan (Ouzzani et al. 2016) for both stages of the search process.

### 2.3 Study Selection

Any type of study which assessed the cost (e.g., Cost‐Effectiveness Analysis, Cost‐Benefit Analysis, Cost‐Utility Analysis and Cost‐Consequences Analyses) of an intervention which was primarily delivered by a speech and language therapist directly or indirectly (e.g., SLT educating parent/carers) was included. For this review the protected professional title of ‘Speech and Language Therapist’ defined by the Health and Care Professionals Council (HCPC) was used (Health and Care Professionals Council 2024). A speech and language therapist is a registered allied health professional holding a recognized professional qualification in SLT (Health and Care Professional Council 2023). SLT interventions include any form of targeted practice tasks or methodologies with the aim of supporting speech, language, communication or swallowing abilities, activities, or participation (Brady et al. 2016). For studies taking place in countries that use alternative terms for ‘Speech and Language Therapist’, for example, ‘Speech Pathologist,’ it was perceived that the intervention was delivered by an equivalent registered professional, and this distinction was coded as part of data extraction. While the existing evidence emphasizes the need for full economic evaluations, this review aimed to include all relevant studies providing cost or cost‐effectiveness data, as we acknowledged the limited evidence in this area and sought to assess both the totality and maturity of economic evaluations within the SLT research.

### 2.4 Data Extraction (Selection and Coding)

A single reviewer conducted all abstract and title screening (JH) with 10% verification by a second reviewer using Rayyan (GC & GJ) (Ouzzani et al., 2016). Full paper screening was carried out by one reviewer (JH, AK, DM, LM, GC, VB, LS or GJ) with 10% verification by a second reviewer (JH, AK, DM, LM, GC, LS or GJ). All reasons for exclusion of studies at full paper screening were documented. The verification procedures at both stages of screening necessitated a Kappa Score indicating agreement within the range of 0.61 to 1.00 (≥ substantial agreement) (McHugh 2012). Where this level was not obtained, successive 10% increments were reassessed until the desired threshold was reached.

Data extraction was undertaken by one reviewer independently using a pre‐piloted form (JH, AK, DM, LM, GC, VB or GJ) (see Supporting Information S1 for data extraction form). Due to the substantial number of studies and limited resources, the 10% verification process for data extraction was unable to be carried out. However, the data items concerning the type of economic evaluation, method of analysis, perspective, and time horizon were all double‐checked following data extraction (VB). The following data items were extracted: Country of study, Town/city, Study type, Year of publication, DOI, Clinical setting, Population, Intervention descriptive type, Comparator (if applicable), Mode of delivery (independent delivery or volunteer‐facilitated SLT interventions), Individual being supported, Descriptive term for Speech and Language Therapist, Perspective (Kim et al. 2020), Time horizon, Outcomes, Measurement and Valuation of resources, and Method of analysis. The perspective was classified based upon the analysis with the broadest perspective within the study (Kim et al. 2020). The time horizon was classified as the maximum period used in any analysis within the study.

The methods of analysis were coded into four main types: Costing study (only assessed the cost of the intervention without evaluating its effectiveness); cost‐consequence analysis (either defined by the authors as a cost‐consequence analysis or defined by the authors as a cost‐effectiveness study but, upon inspection, did not actually combine cost and effectiveness outcomes) (Anton and Lisa 2022; Lau 2017). When a study does not estimate a cost per additional health gain it cannot be classified as a cost‐effectiveness analysis, rather, it could be a cost‐consequence analysis (where costs and effects for the different alternatives are presented separately) (Lau 2017); cost‐effectiveness analysis (which measures the comparative costs of a unit change in a health outcome through a single measure [e.g., cost per life year gained or cost per stroke averted]); and cost‐utility analysis (which is similar to a cost‐effectiveness analysis but encompasses a measure of both quantity [life years] and quality of life compared to the costs incurred [e.g., cost per quality adjusted life year]) (Anton and Lisa 2022; Lau 2017).

### 2.5 Strategy for Data Synthesis

Due to the expected heterogeneity in study design, interventions, and outcomes, a narrative synthesis approach was used to summarize the findings. Study counts per year were presented using bar charts to highlight the growth within this area over time [Q1]. The publication year was determined based on the primary study that reported the economic evaluation, rather than any link study that reported the effectiveness data only. A geographical map of study locations was utilized to identify regional patterns in the studies which assessed the cost or cost‐effectiveness of the SLT intervention (Samarasundera et al. 2012) [Q2]. Litmap's citation mapping software was used to assess the interrelationship of citations within this field and to help review the success of forward and backward citation searching within this area of study (Litmaps 2024) [Q3]. The citation map exclusively utilized the primary paper containing the economic evaluation data to establish the interconnectedness of the evidence base. This approach was chosen to emphasize the interconnectivity among the economic evaluation papers specifically, rather than the broader findings of the effectiveness studies. Counts of studies were assessed by clinical population, study type, clinical setting, mode of delivery and perspective taken (Shields and Elvidge 2020) [Q4 & Q5].

## 3 Results

**FIGURE 1:**
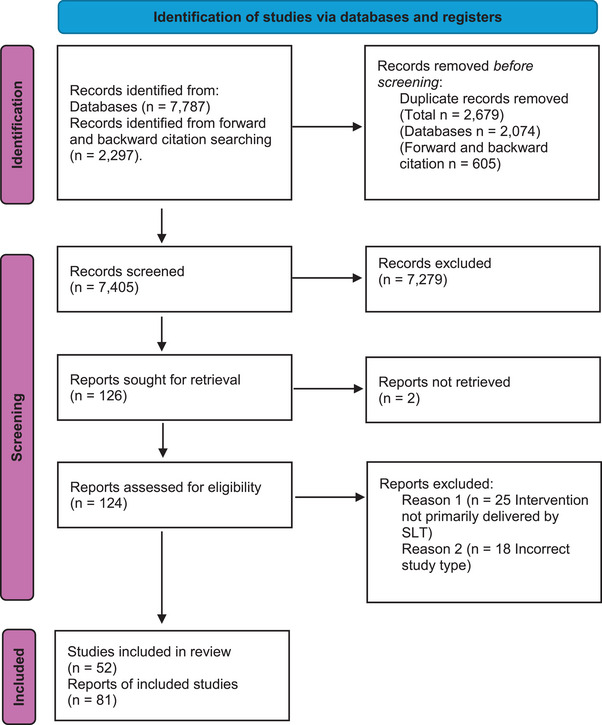
PRISMA flow diagram (Page et al. 2021).

After duplicate removal, a total of 7405 citations were identified from the multi‐database search and forward and backward citation screening. After abstract and title screening, 126 studies were retrieved for full paper evaluation. Following full paper screening, 52 studies (81 citations) met the inclusion criteria and were included in the scoping review (see Figure 1 for PRISMA flow diagram). The Kappa Score for screening abstracts and titles demonstrated substantial agreement among all reviewers, with a mean Kappa Score of 0.67. Likewise, the Kappa Score for screening full papers indicated substantial agreement among all reviewers, with a mean Kappa Score of 0.70. The main reason for exclusion at full paper screening was due to the intervention not being primarily delivered by a speech and language therapist (*n* = 25), followed by the study not reporting the cost or cost‐effectiveness of an SLT intervention (*n* = 18) (see Supporting Information S1: Appendix 3 for a full list of excluded studies). Regarding validation of the primary search strategy, only three additional papers were identified in the forward and backward citation screening, of which one was a link paper and the remaining two were new studies not identified within the primary search strategy.

Out of the 52 studies included, eight were protocol‐only studies (no additional publications with economic evaluation data being published). One of these protocol‐only studies, the PRESTO trial, has started publishing their findings (International Standard Randomised Controlled Trial Number 2018). However, despite stipulations within the protocols for collecting cost‐effectiveness data, no analysis of such data has yet been published. Out of the remaining 44 completed studies, six were published in abstract form only, while the remaining 38 were published as full papers. These six abstracts were included in conference proceedings and provided limited data compared to the full papers.

Q1: How has the volume and type of studies assessing the cost or cost‐effectiveness of SLT interventions evolved over time?

**FIGURE 2:**
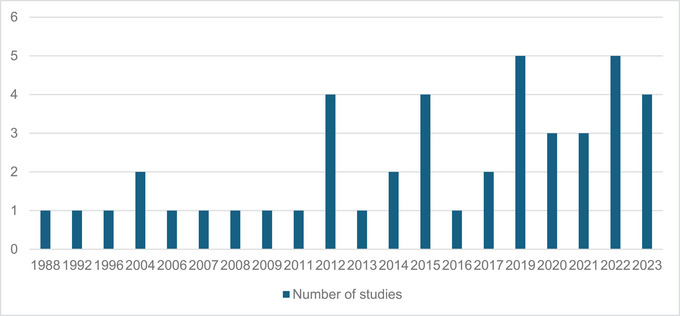
Number of studies which assessed the cost or cost‐effectiveness of SLT interventions published each year.

The earliest study examining the cost or cost‐effectiveness of an SLT intervention was published in 1988 (see Figure 2 for a bar chart of the number of studies examining the cost or cost‐effectiveness of SLT interventions per year). Throughout the subsequent two decades, there were only eight additional studies (18.2%) reporting the cost or cost‐effectiveness of an SLT intervention. However, in the following decade, the number of publications doubled (36.4%, *n* = 16). More recently, since 2019, there has been a notable increase in publications, ranging between 3 to 5 per year (45.5%, *n* = 20). It is also important to note that more than half of the studies were conducted alongside randomized controlled trials (RCTs). Of the 20 studies published since 2019, 15 are either cost‐utility analyses or cost‐effectiveness analyses. Between 2016 and 2023, the remaining eight protocols, for which the cost or cost‐effectiveness data have not been published, were registered, with almost one protocol registered each year.

Q2: What is the geographical distribution of studies assessing the cost or cost‐effectiveness of SLT interventions?

**FIGURE 3:**
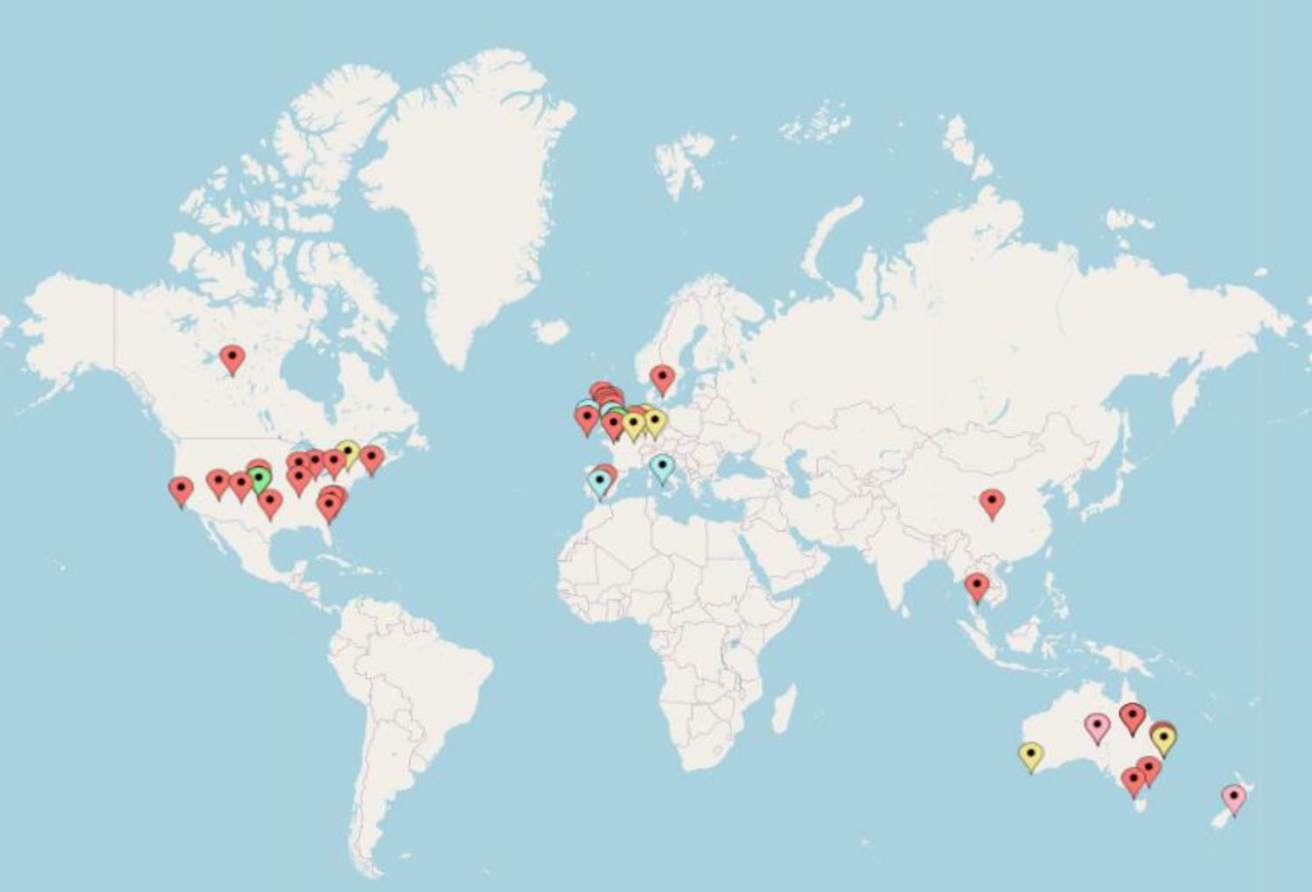
Geographical location of studies which assessed the cost or cost‐effectiveness of SLT interventions. Key: Single study location studies, Multiple study location studies, Protocol only studies.

The majority of completed studies were undertaken in three main regions: the United States of America (31.8%, *n* = 14), Australia (22.3%, *n* = 10) and the United Kingdom (20.5%, *n* = 9) (see Figure 3 for the geographical location of all included studies). The remaining studies were published in Canada (4.5%, *n* = 2), China (2.3%, *n* = 1), Ireland (2.3%, *n* = 1), Netherlands (2.3%, *n* = 1), Spain (2.3%, *n* = 1), Sweden (2.3%, *n* = 1), Thailand (2.3%, *n* = 1), Australia & New Zealand (2.3%, *n* = 1), the United States of America & United Kingdom (2.3%, *n* = 1), and England, Ireland, Italy, and Spain (2.3%, *n* = 1). Of the eight studies with published protocols only, these are planned to be undertaken in Australia (*n* = 2), Germany (*n* = 2), Belgium (*n* = 1), Canada (*n* = 1), Netherlands (*n* = 1) and the United Kingdom (*n* = 1).

Q3: What is the interconnectivity of studies assessing the cost or cost‐effectiveness of SLT interventions within the existing evidence base?

**FIGURE 4:**
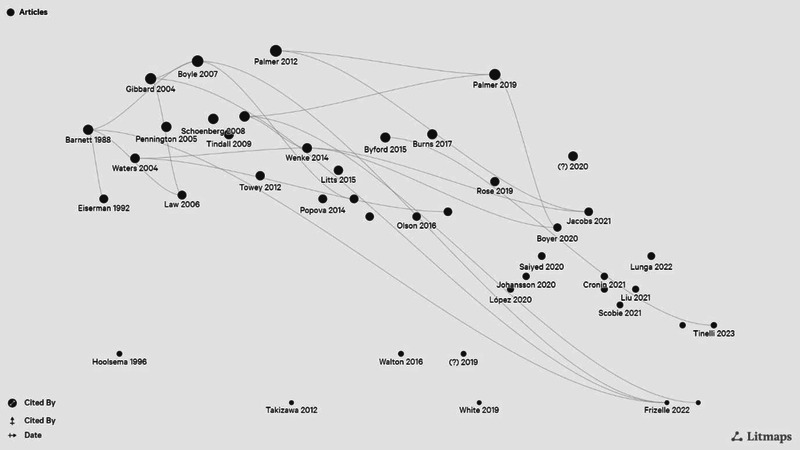
Citation map.

One study (published solely in abstract form within conference proceedings) could not be located using the literature mapping software, with the resulting citation map comprising 43 studies (see Figure 4). Only 18 studies cited another study included in the review. The most cited study was the seminal paper by Barnett et al. (1988) with four citations.

Q4 & Q5: Which clinical populations have been the focus of studies assessing the cost or cost‐effectiveness of SLT interventions, and what are the characteristics of these evaluations?

### 3.1 Clinical Population

Out of the 44 completed studies included in the review, the majority concentrated on economic evaluations of interventions for adults (*n* = 26), followed by those for children (*n* = 17) and mixed populations (*n* = 1).

#### 3.1.1 Adult Stroke Patients

The predominant population of interest across the reviewed studies was adult stroke patients, with nine studies specifically addressing this population. Among these, seven studies explored interventions targeting aphasia, while two studies addressed both aphasia and dysarthria as well as dysphagia. Detailed information regarding the studies assessing the cost or cost‐effectiveness of SLT interventions for adult stroke patients can be found in Table 1.

**TABLE 1:** Study characteristics of studies which assessed cost or cost‐effectiveness of a SLT intervention for adult stroke patients or adult with aphasia or dysarthria.

Study names	Study design	Population of interest	Clinical setting	Intervention type	Comparator (if applicable)	Type of economic evaluation	Perspective	Horizon	Outcomes assessed
Bowen et al. (2012) Link papers: Boyle et al. (2009), Davies (2011), Nct (2005, 2009)	RCT	Adult, Stroke, Aphasia or Dysarthria	Hospital inpatient and outpatients	Enhanced early communication therapy by speech and language therapists	Attention control (equivalent amount of contact time (attention) as those in the intervention arm (up to three times a week, for up to 3 months)	Cost‐utility analysis	Healthcare sector and patients/families	6 months	Functional communicative ability: Measured at 6 months post‐randomization using the Therapy Outcome Measure activity subscale. Perceptions of communication: Assessed through the Communication Outcomes After Stroke scale for participants and part of the Carer COAST for carers. Carer well‐being: Evaluated using the Carers of Older People in Europe Index and quality‐of‐life items from Carer COAST. Economic evaluation: Included participants’ utility measured by the European Quality of Life‐5 Dimensions, service use, cost data, and a discrete choice experiment.
Jacobs et al. (2023) Link paper: Jacobs and Ellis (2021)	Before and after study	Adult Stroke Aphasia	Community	Community‐based telerehabilitation approach [Language‐Oriented Treatment] [telehealth]	No control group. Study was focused on comparison by aphasia subtype but also compared outcomes by severity and race.	Cost‐effectiveness analysis	Healthcare payer	6 week	Aphasia treatment benefit: The therapeutic benefit was measured using the Western Aphasia Battery‐Revised Aphasia Quotient before and after telerehabilitation treatment. Marginal cost of treatment: The marginal cost was calculated based on the change in Western Aphasia Battery‐Revised Aphasia Quotient and the average cost per treatment session. Cost‐effectiveness by aphasia type: The study evaluated the cost‐effectiveness of treatment by comparing the improvement in Western Aphasia Battery‐Revised Aphasia Quotient scores relative to the type and severity of aphasia.
Kim et al. (2023) Link papers: Godecke et al. (2014), Godecke et al. (2013) Godecke et al. (2015), Godecke et al. (2017), Godecke et al. (2016), Godecke et al. (2014), Godecke et al. (2018), Kim et al. (2021)	RCT	Adult Stroke Aphasia	Hospital inpatient and outpatients	Very Early Rehabilitation in Speech (VERSE) intervention Usual Care Plus: usual ward‐based therapy and 20 additional sessions (45–60 min, provided daily)	Usual care	Cost‐effectiveness	Societal perspective	26 weeks post‐stroke	Costs: Estimation of costs for patients with aphasia after stroke based on the therapies provided. Healthcare utilization: Analysis of healthcare resources used and productivity losses. Aphasia severity: Measurement of clinically meaningful change in aphasia severity using the Western Aphasia Battery‐Revised Aphasia Quotient. Quality of life: Comparison of the Stroke and Aphasia Quality of Life Scale‐39 scores by study arm and baseline aphasia severity.
Liu et al. (2021)	RCT	Adult Stroke Aphasia	Community	Acupuncture therapy combined with speech and language therapy	Speech and language therapy alone	Cost utility analysis	Societal perspective	12 weeks	BDAE: Boston Diagnostic Aphasia Examination grades. CRRCAE: Chinese Rehabilitation Research Center Standard Aphasia Examination scores. QALYs: Quality‐adjusted life‐years. ICER: Incremental cost‐effectiveness ratios for BDAE grade improvement, CRRCAE score gain, and QALYs gained.
Palmer et al. (2019) Link papers : Latimer et al. (2021), Palmer et al. (2015), Palmer et al. (2020), Palmer et al. (2011)	RCT	Adult Stroke Aphasia	In the service user's home	(1) Self‐managed computerized (StepByStep aphasia software) speech and language therapy [telehealth]	(2) Usual care (Assessment and review of language abilities and their impact, rehabilitation of different language domains, enabling communication using communication aids or compensatory strategies, or support for mood, confidence, work, family, form completion, and information provision.) (3) Usual care + attention control (Puzzle books (Sudoku, spot the difference, mazes, word searches, cross words, colouring)).	Cost utility analysis	v Healthcare sector	Lifetime time horizon	Word finding ability: The change in the ability to retrieve personally relevant words was measured using a picture naming test. Functional communication: The change in functional communication ability was assessed by masked ratings of video‐recorded conversations using the Therapy Outcome Measures. Self‐perception: The change in patients’ self‐perception of communication, social participation, and quality of life was measured using the Communication Outcomes After Stroke questionnaire. Health‐related quality of life: This was measured using an accessible variant of the European Quality of Life. Instrument (EQ‐5D‐5L) for patients, and standard EQ‐5D‐5L completed by carers on behalf of patients. Carers also completed the CarerQoL questionnaire to assess their own quality of life. Adverse events compared.
Palmer et al. (2012)	RCT	Adult Stroke Aphasia	In the service user's home	Self‐managed computerized speech and language therapy [telehealth]	Usual care (participation in activities that provide general language stimulation as they had done previously: attendance at communication support groups and conversation, reading, and writing activities that are part of everyday life)	Cost utility analysis	Health and social care system	Lifetime horizon	Recruitment and completion rates: The study aimed to determine the feasibility of recruiting and retaining participants for a randomized controlled trial. Clinical effectiveness: The effectiveness of the computer therapy was measured by the change in word retrieval ability at 5 and 8 months from baseline. Cost‐effectiveness was investigated by estimating total costs and total quality‐adjusted life‐years, incremental cost‐effectiveness ratio. Intervention Feasibility: The study evaluated the ability of participants to carry out the self‐managed intervention as prescribed.
Rose et al. (2019) Link papers :Rose et al. (2015), Rose et al. (2021), Rose et al. (2022)	RCT	Adult Stroke Aphasia	Community	1. Multi‐Modal Aphasia Treatment [M‐MAT] (3 h multi‐modality group therapy, 5 days a week for 2 weeks) 2. Constraint‐Induced Aphasia Therapy Plus [CIAT‐Plus]: (3 h constraint‐induced group therapy, 5 days a week for 2 weeks)	Usual care service‐based aphasia therapy (Care as per usual in the community: estimated at < 2 h/week)	Cost‐utility analysis	Healthcare system and patients	12 weeks	Primary outcome: The primary measure was the Western Aphasia Battery‐Revised Aphasia Quotient, assessed immediately after the intervention. Secondary outcomes: These included the Western Aphasia Battery‐Revised Aphasia at 12‐week follow‐up, naming scores, discourse measures, the Communicative Effectiveness Index, the Scenario Test, and the Stroke and Aphasia Quality of Life Scale‐39 g both immediately and at 12 weeks post‐intervention. Economic evaluation: The study also looked at incremental cost‐effectiveness ratios compared with usual care at 12 weeks.
Takizawa et al. (2012)	Costing study	Adult Stroke Dysphagia	Acute and inpatient	Swallowing disorder rehabilitative session	Managed dysphagia vs. unmanaged dysphagia	Costing study	Healthcare payer	Not clear	Cost: The cost of speech and language therapy (unit costs per patient).
Wenke et al. (2014)	Non‐randomized controlled trial	Adult Stroke Aphasia	Hospital inpatient and outpatients	Intensive communication therapy by speech and language therapists (additional 1–1.5 h of treatment employing either the use of 1. Computer therapy (software programs including REACT‐2, Aphasia Tutor, Language Links, and Synonyms) 2. Group therapy (Four‐to‐six participants participated in each group therapy session which was facilitated by a speech and language therapist)	Speech pathology therapy assistant therapy (The tasks provided by the speech pathology therapy assistant were planned by the treating speech and language therapist and reflected similar tasks that participants received during their individual speech and language therapy)	Consequence analysis*	Healthcare sector	Not clear	Spoken language production: This included sub‐tests for naming objects, naming actions, and word fluency. Disability Questionnaire: A standardized questionnaire measuring the impact of aphasia on daily life and emotional well‐being. Organizational outcomes: Data collected to determine the costs associated with each service delivery model. Cost of service: Pro‐rata cost of providing treatment per hour per client. Client, caregiver, and clinician satisfaction: Questionnaires evaluated the satisfaction levels of all parties involved with the intensive
Dowlatshahi et al. (2019)	Protocol: RCT	Adult Stroke	Acute and inpatient	Mobile tablet‐based speech therapy [telehealth]	Usual care (speech and language therapist)	Cost‐Utility Analysis	Not clear	Not clear	Change in Western Aphasia Battery scores Cost‐effectiveness incremental cost per one‐unit improvement in AQ & incremental cost per one quality‐adjusted life year. Stroke and Aphasia Quality of Life Scale Communicative Effectiveness Index Cognitive Linguistic Quick Test‐Plus The 5‐level EQ‐5D version National Institutes of Health Stroke Scale Barthel index Modified Rankin Scale
Spielmann et al. (2016)	Protocol: RCT	Adult Stroke Aphasia	Acute and inpatient	Transcranial direct current stimulation	Sham treatment (activated for 30 s and then deactivated to mimic the sensation of active tDCS without providing actual stimulation)	Cost utility analysis	Societal (productivity costs included)	6 months	Primary outcome: The primary focus was on word‐finding abilities, measured by the Boston Naming Test. Secondary outcomes: These included measures of verbal communication, social participation, quality of life, and the cost‐effectiveness of the intervention, Wong‐Baker Faces pain rating scale. Verbal communication: Aphasia Severity Rating Scale and the Amsterdam Nijmegen Everyday Language Test. Quality of life and participation: Assessed using the European quality of life‐5D, Stroke and Aphasia Quality of Life questionnaire, and the Community Integration Questionnaire.
Stahl et al. (2019)	Protocol: RCT	Adult Stroke Aphasia	Acute and inpatient	Transcranial direct current stimulation	Sham treatment (mimicked the initial tingling sensation of actual transcranial direct current stimulation)	Cost‐utility analysis	Healthcare system and patients	12 months	Primary outcome: The main focus was on communication ability, measured by changes in the Amsterdam‐Nijmegen Everyday Language Test scores. Secondary outcomes: These included assessments of linguistic‐executive skills, attention, memory, emotional well‐being, quality of life, health economic costs, and adverse events.

*Note*: * Defined by the authors as a cost‐effectiveness study but did not actually combine cost and effectiveness outcomes. Grey area indicates a protocol for a study.

Abbreviations: BDAE—Boston Diagnostic Aphasia Examination grades; CarerQoL—Carer Quality of Life; COAST—Carer Communication Outcome after Stroke carer communication; CRRCAE—Chinese Rehabilitation Research Center Standard Aphasia Examination scores; ICER—Incremental Cost‐Effectiveness Ratio; QALYs—Quality‐Adjusted Life Years, RCT—Random controlled trial.

The research was conducted across various clinical settings, including community settings (*n* = 5), hospital inpatient and outpatient facilities (*n* = 3), and hospital inpatient settings exclusively (*n* = 1). Assessment of SLT costs or cost‐effectiveness was integrated into different study designs, encompassing RCTs (*n* = 6), non‐randomized controlled trials (*n* = 1), before‐and‐after studies (*n* = 1), and costing studies (*n* = 1). The interventions implemented in these studies exhibited considerable diversity, with no standardized intervention types. However, certain intervention themes emerged: the use of telehealth (*n* = 3) and ‘early’ SLT (*n* = 2). There was variation in the types of economic evaluation used, with three primary analytical approaches observed: cost‐utility analysis (*n* = 5), cost‐effectiveness analysis (*n* = 2), cost consequence analysis (*n* = 1), and costing study (*n* = 1). These analyses were conducted from multiple perspectives, including societal (*n* = 2), healthcare sector (*n* = 2), healthcare payer (*n* = 2), healthcare sector and patients/families (*n* = 2) and health and social care system (*n* = 1). Furthermore, the time horizons varied significantly across studies, with time horizons ranging from 6 months to the participants’ lifetime. Additionally, three RCT protocols have been registered which indicate cost outcomes within this population, although as of now, no cost results have been published. It is encouraging to note that all three studies are aiming to conduct cost‐utility analyses. However, detailed information regarding the extent of the analysis methods is limited. Full study characteristics for protocols are reported in Table 1.

#### 3.1.2 Adults With Aphasia or Dysarthria

There were also four studies which assessed the cost or cost‐effectiveness of SLT interventions for adults with aphasia (*n* = 2) and dysphagia (*n* = 2). Whilst both are often related to stroke (National Institute for Health and Care Excellence 2023a), no underlying aetiology was identified in these studies. The assessment of the cost or cost‐effectiveness of SLT interventions was conducted within the framework of a non‐randomized controlled trial (*n* = 2) or a retrospective observational study (*n* = 2). Similarly, these studies exhibited considerable variation in intervention approaches. Detailed information regarding the studies assessing the cost or cost‐effectiveness of SLT interventions for adults with aphasia or dysarthria can be found in Table 2.

**TABLE 2:** Study characteristics of studies which assessed cost or cost‐effectiveness of a SLT intervention for adult with aphasia or dysarthria.

Study names	Study design	Population of interest	Clinical setting	Intervention type	Comparator (if applicable)	Type of economic evaluation	Perspective	Horizon	Outcomes assessed
Boyer et al. (2022)	Retrospective observational study	Adults with Aphasia	Acute and inpatient	Intensive Comprehensive Aphasia Programme	None (although running the programme again after the initial intervention was compared)	Costing study	Healthcare payer	4 weeks	Total implementation cost: The primary outcome measured was the total cost to the provider for implementing an Intensive Comprehensive Aphasia Program. Personnel costs: A significant portion of the costs was attributed to personnel, especially the time of the speech language pathologist. Break‐even charges: The study analysed break‐even charges per participant, which varied based on the number of participants. Cost drivers: The main cost drivers identified were personnel costs and the number of participants in each cohort.
Burns et al. (2019)	Non‐randomized controlled trial	Adults with Dysphagia	Outpatient	Telepractice service model for conducting clinical swallow examinations implemented [telehealth]	Standard care, which involved scheduled/on‐demand clinician visits to remote services or patients travelling to face‐to‐face assessment	Consequence analysis*	Healthcare payer	One off assessment	Waiting times: number of days Clinical session outcomes: no specific tool was described Service costs: total cost Consumer satisfaction: no specific tool was described
Hobson et al. (2013)	Non‐randomized controlled trial	Adults with Aphasia	Hospital inpatient	(1) Computer therapy [telehealth] (2) group therapy.	(3) Use of speech pathology therapy assistant	Consequence analysis*	Healthcare sector	11 weeks	Patient language function: no specific tool was described Attendance: no specific tool was described Cost of intervention: pro rata cost of intervention per hour
Thomas et al. (2020)	Retrospective observational study	Adults with Dysphagia	Acute and inpatient	Modified Barium swallow	Fibreoptic endoscopic	Consequence analysis*	Healthcare payer	One year	Discharge disposition: The primary outcome measured is where patients were discharged to after their stay at the inpatient rehabilitation facility, particularly whether they were discharged home or to another type of facility. No specific description of the outcome for cost evaluation.

*Note*: * Defined by the authors as a cost‐effectiveness study but did not actually combine cost and effectiveness outcomes.

All the included studies conducted a cost consequence analysis (*n* = 3) or were a costing study without integrating any form of effectiveness data (*n* = 1). The time horizon varied, ranging from 4 weeks to 1 year, with perspectives predominantly focused on either healthcare payer (*n* = 3) or healthcare sector (*n* = 1) viewpoints.

#### 3.1.3 Adult Head and Neck Cancer Patients

Four studies investigated the cost or cost‐effectiveness of SLT interventions for adults undergoing treatment for head and neck cancer (see Table 3 for full study characteristics). The economic evaluations were undertaken alongside an RCT (*n* = 3) or a feasibility study (*n* = 1). All studies took place in outpatient settings. Interventions were all enhanced versions of usual care with additional SLT delivery.

**TABLE 3:** Study characteristics of studies which assessed cost or cost‐effectiveness of a SLT interventions for adult head and neck cancer patients.

Study names	Study design	Population of interest	Clinical setting	Intervention type	Comparator (if applicable)	Type of economic evaluation	Perspective	Horizon	Outcomes assessed
Burns et al. (2017)	RCT	Adult cancer, including head and neck cancer	Outpatient	Patient continuing to attend SLT appointments regionally but with specialist SLT support via telehealth with patient present)	Usual care: (attend appointments at their regional hospital with their local speech pathologist, while specialist support for the referred problem was provided by the Royal Brisbane and Women's Hospital speech pathologist to the regional speech pathologist predominantly without the patient present, via email/telephone contact when convenient and clinically indicated))	Cost consequence analysis*	Healthcare payer	11 months	Health service costs: Calculated based on staff wages, equipment, patient travel reimbursement, and other service‐related expenses. Patient & carer costs: Included travel expenses, wages lost due to treatment, and quality of life impacts measured by the Assessment of Quality‐of‐Life questionnaire 4D. Quality of life: Utilized the Assessment of Quality‐of‐Life questionnaire 4D to measure changes in health‐related quality of life for patients.
Johansson et al. (2020)	RCT	Adult cancer, including head and neck cancer	Outpatient	Voice rehabilitation	Usual care (general vocal hygiene advice according to clinical practice)	Cost utility analysis	Societal perspective	12 months	Voice rehabilitation efficacy: The effectiveness of voice rehabilitation post‐radiotherapy. Quality‐Adjusted Life Years: QALYs Healthcare costs: Direct healthcare costs and loss of production were analysed to determine the cost‐effectiveness of voice rehabilitation. Cost‐effectiveness analysis: The incremental cost‐effectiveness ratio was calculated to compare voice rehabilitation with no rehabilitation intervention.
Martino et al. (2017) Link paper: Martino et al. (2015)	Feasibility study	Adult cancer, including head and neck cancer	Outpatient	Mostly face to face, some follow ups completed via telephone	Standard care	Cost consequence analysis*	Patients and caregivers	3 months	Delay to removal of an enteral feeding tube after completion of treatment. M.D. Anderson Dysphagia Inventory. The Functional Assessment of Cancer. Therapy–Enteral Feeding. Swallow Quality of Care questionnaire. European quality‐of‐life Research. Foundation, Rotterdam, Netherlands. Functional Oral Intake Scale. Body Mass Index. Patient Self‐Administered Financial. Expenditure.
Waters et al. (2004)	RCT	Adult cancer, including head and neck cancer	Outpatient	Swallowing rehabilitation intervention strategy (swallowing exercises 16 × 1 h sessions)	Waiting‐list control	Cost consequence analysis*	Patient and health care trust	8 months	Oral transit time. Pharyngeal transit time. Duration of tongue base retraction. Duration of tongue base to pharyngeal wall contact at the level of inferior C2. Duration of tongue base to pharyngeal wall contact at the level of superior C3. Pharyngeal delay time. Pharyngeal response time. Duration of hyoid movement. Duration of laryngeal elevation. Oral transit time. Pharyngeal transit time. Duration of tongue base retraction. Duration of tongue base to pharyngeal wall contact at the level of inferior C2.
International Standard Randomised Controlled Trial Number (2023)	Protocol: Feasibility study	Adult cancer, including head and neck cancer	Outpatient	60‐min outpatient speech and language therapy appointment once a week for 6 weeks	Usual care	Cost‐utility analysis	Healthcare payer and patient	18 months follow‐up	Pressure measurements: Using high‐resolution manometry at baseline and 3 months. Repeat swallows: Number of repeat swallows measured with the 100 mL water swallow test. Swallow Score: Patient report of swallow score using the MD Anderson Dysphagia Inventory. Pharyngeal constriction: Ratio measurement using video fluoroscopy swallow evaluation. Diet Level Score: Clinician report using the Functional Oral Intake Scale. Neck range of motion: Score measured with a goniometer. Cost‐effectiveness: Assessed using the EuroQol EQ‐5D‐5L health‐related quality of life measure.
International Standard Randomised Controlled Trial Number (2018)	Protocol: RCT	Adult cancer, including head and neck cancer	Outpatient	Patients referred to Group 1 or 2 will practice at home following an instruction session. Group 2 will receive an additional app to support in this delivery.	Receives speech therapist supervised therapy	Cost‐utility analysis	Not explicitly stated	4 months	Swallowing function: Assessed using the Mann Assessment of Swallowing Ability—Cancer, Eating Assessment Tool (EAT‐10), a visual analogue scale for self‐perception of swallowing ability, and the Functional Oral Intake Scale 50. Compliance: The degree of adherence to the exercise program is measured weekly through patient and therapist logbooks, the IOPI device for tongue strengthening exercises, and app usage data for group. Muscle strength: Tongue strength is measured using the Iowa Oral Performance Instrument, and maximum muscle strength during Chin Tuck Against Resistance is assessed with a dynamometer. Quality of life: Evaluated using the Swallowing Quality of Life Questionnaire and the Dysphagia Handicap Index.

*Note*: * Defined by the authors as a cost‐effectiveness study but did not actually combine cost and effectiveness outcomes. Grey area indicates a protocol for a study.

Abbreviations: QALYs—Quality‐Adjusted Life Years; RCT—Randomized controlled trial.

Most of the studies only conducted a cost consequence analysis, directly comparing the costs associated with the enhanced interventions to those of usual care (*n* = 3). Only one study performed a cost‐utility analysis. Various perspectives were adopted in these analyses, including healthcare payer (*n* = 1), limited societal (*n* = 1), patients and caregivers (*n* = 1) and societal perspective (*n* = 1). The time horizon for the evaluation in these studies ranged from 3 to 12 months.

In addition to these completed studies, two ongoing protocols were registered to investigate the cost‐effectiveness of SLT interventions for adults with head and neck cancer. One of these studies is a feasibility study for an RCT, while the second is an RCT. Both studies are planned to be conducted within an outpatient setting. These two studies employ different approaches, with one utilizing a 60‐min outpatient appointment and the other employing varying modes of delivery. The planned economic evaluations aim to incorporate a cost‐utility analysis, with one study specifying the use of a healthcare payer and patient perspective and one study not explicitly defining the perspective. The time horizons for these planned economic evaluations range from 4 to 18 months.

#### 3.1.4 Adults With Parkinson's Disease

Three studies evaluated the cost or cost‐effectiveness of SLT interventions for adults with Parkinson's disease. Two of these focused on interventions for people with Parkinson's disease and one on interventions for carers. Two of the economic evaluations were run alongside RCTs, while one study solely conducted a costing study without evaluating effectiveness. These studies were conducted either in an outpatient (*n* = 2) or community (*n* = 1) setting (see Table 4 for full study characteristics).

**TABLE 4:** Study characteristics of studies which assessed cost or cost‐effectiveness of a SLT interventions for adults with Parkinson's disease.

Study names	Study design	Population of interest	Clinical setting	Intervention type	Comparator (if applicable)	Type of economic evaluation	Perspective	Horizon	Outcomes assessed
Saiyed et al. (2022)	RCT	Adults with Parkinson's disease	Outpatient	One‐to‐one speech and language 1 h a day for 4 weeks (LSVT LOUDVR programme) video‐linked [telehealth] (urban group and regional group)	One‐to‐one speech and language 1 h a day for 4 weeks (LSVT LOUDVR programme) face‐to‐face	Cost consequence analysis*	Healthcare system and patients	1 month	Cost analysis: Comparison of costs between in‐person speech treatment and home telerehabilitation Health‐system perspective: Evaluation of treatment costs from a health‐system viewpoint. Patient perspective: Analysis of treatment costs from the patient's perspective, including travel and income loss. Speech outcomes: The study utilized acoustic and perceptual measures to evaluate speech outcomes. Quality‐of‐life Outcomes: The study assessed the impact of the treatment on patients’ quality of life.
Scobie et al. (2021) Link paper: Sackley et al. (2016), Sackley et al. (2020)	RCT	Adults with Parkinson's disease	Outpatient	(1) Lee Silverman Voice Treatment (2) Standard speech and language therapy	(3) No intervention in first 6 months, unless deemed medically necessary	Cost utility analysis	Healthcare sector	12 months	Voice Handicap Index (VHI): A measure of the psychological impact of voice disorders. Parkinson's Disease Questionnaire‐39 (PDQ‐39): A quality of life measure specific to Parkinson's disease, including a summary index and communication domain score. EuroQoL EQ‐5D‐3L: A standardized instrument for measuring general health status. ICECAP‐O: A measure of capability for older adults, covering attributes like attachment, security, role, enjoyment, and control. Cost outcomes: course assessment was carried out based upon cost to the NHS and cost of the intervention. The cost utility unit used in the study was the EQ‐5D‐3L.
Tindall and Huebner (2009)	Costing study	Adults with Parkinson's disease (caregivers)	Community	1:1 Telehealth and some face to face [telehealth]	Traditional delivery model where caregivers travel to the clinic for therapy.	Costing study	Caregivers	1 month	Time and financial savings: Time and financial obligations for caregivers. Caregiver burden: Physical, emotional, and financial toll of providing care.

*Note*: * Defined by the authors as a cost‐effectiveness study but did not actually combine cost and effectiveness outcomes.

All three studies investigated various modes of delivery compared to alternative methods (active comparison). One study produced a costing study, one study undertook a cost consequence analysis, and one study undertook a cost‐utility analyses. These assessments were conducted over a time horizon ranging from 1 month to 12 months. The studies used a either a healthcare system and patients or healthcare sector and caregivers.

#### 3.1.5 Adults With Varying Conditions

Seven studies (*n* = 7) and one protocol presented economic evaluations assessing a range of SLT interventions (e.g., voice therapy, telehealth, generic SLT) in adults with differing conditions (e.g., tracheostomy, voice conditions, geriatric, respiratory) (See Table 5 for full study characteristics). The economic evaluations were conducted alongside an RCT, a non‐randomized trial and six observational studies. Most studies were cost consequence analyses (*n* = 5), with only two cost‐effectiveness analyses and one cost‐utility analysis produced. Studies were either from a healthcare payer (*n* = 6) or a societal (*n* = 2) perspective, taking time horizons from 1 to 18 months.

**TABLE 5:** Study characteristics of studies which assessed cost or cost‐effectiveness of a SLT intervention for adults with a range of conditions.

Study names	Study design	Population of interest	Clinical setting	Intervention type	Comparator (if applicable)	Type of economic evaluation	Perspective	Horizon	Outcomes assessed
Hoolsema (1996)	Before and after study	Adults with non‐specific condition or problem	Acute and inpatient	Speech therapy	None	Cost‐Effectiveness Analysis	Healthcare payer	9 months	Functional communication measure: designed by American Speech‐Language‐Hearing Association taskforce on treatment outcome Cost: Total cost of speech and language therapy intervention
Litts et al. (2015)	Retrospective observational study	Adults with a Voice ‐related issues	Outpatient	Voice therapy	Study compared patients evaluated by a laryngologist and Speech and Language Therapist against control group assessed by laryngologist only	Cost consequence analysis*	Healthcare sector	3 months	Therapy attendance: The study measured the number of therapy sessions attended and the number of cancellations or no‐shows Voice therapy outcomes: Changes in Voice Handicap Index‐10 scores Discharge Reasons: The reasons for patients being discharged from therapy, whether they met therapeutic goals or not Cost: The study examined the financial repercussions of co‐assessment, including potential revenue lost due to missed appointments and the effect on SLP billing revenue
Lunga et al. (2022)	Retrospective observational study	Respiratory care in adults	Unclear	Speech therapy	Costs for patients who initiated versus did not initiate speech therapy and who had successful versus unsuccessful therapy were compared	Cost consequence analysis*	Societal	12 months	Time to diagnosis: The duration from the onset of dyspnoea symptoms to the diagnosis of paradoxical vocal fold movement Healthcare costs: Direct and indirect costs incurred before and after the diagnosis, including office visits, procedures, and prescribed pharmaceuticals Lost wages: Indirect costs associated with lost wages due to healthcare visits Treatment outcomes: The dichotomy of therapy outcomes into successful (significant symptom improvement) and unsuccessful (persistent or worsened symptoms)
Mills et al. (2019)	Before and after study	Adults with a Tracheostomy	Critical care	Assessed by speech and language therapist and patients only eating and drinking with the cuff inflated if found to be safe	Patients prior to implementation of change	Cost consequence analysis*	Healthcare payer	10 months	Length of stay: intensive care unit length of stay & Ward length of stay Cost: Cost savings Total mortality for hospital stay Number of chest x‐rays on intensive care unit
Sanz Lopez et al. (2020)	RCT	Adults with a Voice ‐related issues	Outpatient	Standard SLT (traditional supervised speech therapy sessions)	Tube phonation (performed exercises involving phonating into water through a tube)	Cost‐Effectiveness	Healthcare payer	1 year	GRBAS Scale: The subjective evaluation of patients’ voices using the GRBAS scale Cost‐effectiveness: Analysis of the healthcare costs associated with Tube phonation and Standard SLT treatments
Schoenberg et al. (2008)	Non‐randomized controlled trial	Adults with a Brain injury	Outpatient	Computer‐based cognitive teletherapy rehabilitation [telehealth]	Face‐to‐face speech–language rehabilitation (rehabilitation was a programmatic outpatient speech and cognitive therapy program delivered in a face‐to‐face manner by certified and licensed speech–language therapists who had a minimum of 10 years’ experience)	Cost consequence analysis*	Healthcare sector	6 months	Independent living: Determining if participants required in‐home care Independent driving: Assessing if participants could pass a driving course or the state's driving examination. Return to work/school: Evaluating if participants engaged in paid or volunteer work or attended school classes for more than 31 h per week Hours of therapy: The total number of hours participants engaged in either teletherapy or face‐to‐face therapy Cost: total cost of the treatment and a measure of service costs per hour
Towey (2012)	Retrospective observational study	Mixed (adults and children) with vocal cord dysfunction	Community	1:1 Telehealth and some face to face [telehealth]	N/A	Cost consequence analysis*	Healthcare sector	1 month	Cost: cost savings
Payten et al. (2022)	Protocol: Prospective observational cohort study	Adults with non‐specific condition or problem	Outpatient	Speech–language pathology primary contact telehealth [telehealth]	N/A	Cost utility analysis	Limited societal	18 months	Case history information: Case history questionnaire Voice aerodynamic measures of maximum phonation time in seconds and S/Z ratio Perceptual voice quality measures using the Consensus Auditory–Perceptual Evaluation of Voice Acoustic voice quality measures Voice‐ related quality of life: Voice Handicap Index‐ 10 Self‐ reported symptoms of laryngopharyngeal reflux: Reflux Symptom Index Self‐ reported symptoms of laryngeal hypersensitivity: Newcastle Laryngeal Hypersensitivity Questionnaire Health‐related quality of life scores measured using the validated Assessment of Quality of Life‐ 6D Diagnostic classification impression after speech–language pathology primary contact telehealth and laryngoscopy

*Note*: * Defined by the authors as a cost‐effectiveness study but did not actually combine cost and effectiveness outcomes. Grey area indicates a protocol for a study.

Abbreviations: GRBAS—grade, roughness, breathiness, asthenia, strain; RCT—Randomized controlled trial.

#### 3.1.6 Children and/or Young People

Seventeen studies focused on the cost or cost‐effectiveness of SLT services or interventions for children and/or young people. Analysis of the results is constrained by heterogeneity and/or a lack of specificity in reporting, including study population and intervention (see Table 6 for details of study characteristics).

**TABLE 6:** Study characteristics of studies which assessed cost or cost‐effectiveness of a SLT intervention for children and/or young people with a range of conditions.

Study names	Study design	Population of interest	Clinical setting	Intervention type	Individual being supported if applicable	Comparator (if applicable)	Type of economic evaluation	Perspective	Horizon	Outcomes assessed
Prathanee (2011)	Before and after study	Cleft lip and palate (Speech), Early years and school, 3 to 13 years (majority 7–13 years)	Outpatient	Speech therapy provided by five speech and language pathologists, including individual and group therapy, for a total of 18 h during the four‐day speech camp and 6 h in the one‐day follow‐up session.	Children and caregivers	Funding support for the 4‐day speech camp and 1‐day follow‐up were investigated for a comparison of the expenses by individuals with Cleft lip and palate for services from the nearest and only speech centre (Speech Clinic, Srinagarind Hospital, Khon Kaen Province) in northeast of Thailand.	Cost consequence analysis*	Limited societal	6 months	Reduction of articulation errors: during the main speech camp and the one‐day follow‐up session Knowledge: Basic knowledge related to cleft lip and/or palate Cost: Funding support (health service) and expenses (patient) for speech camps
de Sonneville‐Koedoot et al. (2015)	RCT	Stammering Early years and school, 3.0 to 6.3 years	Outpatient	The Lidcombe Program intervention consists of two stages: Stage 1 with a median of 11 to 15 clinic visits and Stage 2 with at least 7 to 12 treatment sessions. The RESTART‐DCM treatment involves weekly clinic visits with a mean of 12 treatment sessions, as per the pilot study.	N/A	Speech and language therapy based on the Demands and Capacities Model (12 sessions)	Cost‐utility analysis	Societal perspective	8 months	Number needed to treat for one patient not to stutter at 18 months Decreased health related quality of life: EuroQoL EQ‐VAS Health Utility Index‐3 Quality adjusted life years (V‐QALYs & U‐QALY) Cost–effectiveness ratio (one additional child who did not stutter at 18 months and Total cost
King et al. (2019)	Before and after study	Oropharyngeal dysphagia Age unspecified	Outpatient	Videofluoroscopic Swallow Study training and mentoring program utilising a remote specialist speech and language therapist attending the Videofluoroscopic Swallow clinic via real‐time synchronous telehealth. [telehealth]	Parents	None	Costing study	Not stated	Not stated	Percentage of families who would prefer their treatment to be delivered either via telehealth or at the clinic
Raatz et al. (2023)	Cost minimisation	Eating/drinking/feeding disorder Early years and school, < 10 years old, majority under 2 years	Outpatient	Tele‐practice (using videoconferencing) paediatric appointments [telehealth]	Parents	Face‐to‐face paediatric appointments	Cost consequence analysis*	Societal perspective	12 months	Cost savings: Per appointment for families Service costs: The health service costs were equivalent for both models, as the clinician's time remained the same for both tele‐practice and in‐person appointments
Finestack et al. (2022)	Feasibility study	Proactive service for children with galactosemia Early years, Starting at < 6 months	Community	Babble Boot Camp (BBC) (Individualized sessions for parents to provide strategies to support their child's communication development) [telehealth]	Parents	None	Costing study	Healthcare system and families	Period of sessions (based on individuals, with minimum of 67 sessions)	Parent satisfaction Survey: 5‐point Likert rating scales Intervention session logs: The study tracked session attendance and modality (Zoom or email) Fidelity checks: Videos were reviewed for adherence to key intervention components (review, teach, model, plan)
Boyle et al. (2007) Link paper: Dickson et al. (2009)	RCT	Language disorder School Primary (6 to 11 years)	Outpatient	(1) Direct individual therapy [speech and language therapist working individually with a child], (2) indirect individual therapy [speech and language therapy assistant (SLTA) working individually with a child]	N/A	(3) Direct group therapy (Speech and Language Therapist working with a small group of children)	Cost‐Effectiveness Analysis	Teaching setting	3.5 months	Language outcome: Standardized scores on tests of expressive and receptive language (CELF‐3) Vocabulary: standardized scores on the BPVS II Parental and teacher observational rating scales linked to the CELF‐3 Binary outcome measure: showed progress postintervention/did not show progress postintervention Enderby's Therapy Outcome Measures (TOM),151 selected to provide standardized information about change of case status Qualitative data: questionnaire, focus group data from parents, teachers, project Speech and Language Therapists and speech and language therapy assistants
Olson et al. (2016)	Before and after study	Language, speech sound or other developmental difficulty affecting speech and language Early years, 11–26 months	Community	Training/education: Text messages to deliver developmental education to families [telehealth]	Parents	Not applicable	Costing study	Healthcare payer	3 months	Program completion rate Response rate to intraporal text messages Parental survey responses to questions (Likert Scale)
Byford et al. (2015)	RCT	Neurodevelopmental conditions, including autism and ADHD (Social communication) Early years, 2 to 5 months	Outpatient	The intervention was the PACT (Pre‐school Autism Communication Trial) therapy, which was a parent‐mediated, communication‐focused intervention delivered by specially trained speech and language therapists. It consisted of fortnightly one‐to‐one clinic sessions for 6 months, followed by monthly booster sessions for an additional 6 months, aiming to target social interactive and communication impairments in children with autism.	Parent	Usual care	Cost‐Effectiveness Analysis	Limited societal	13 months	Autism symptom severity: The severity of autism symptoms was measured using the ADOS‐G social communication algorithm score Parent‐Child Interaction: Video‐rated parent‐child interaction during naturalistic play was assessed Child language and social communication: The study evaluated child language and social communication using the researcher‐assessed Preschool Language Scales Adaptive functioning in school: The Vineland Adaptive Behaviour Scales, Teacher Rating Form, rated by face‐to‐face interview with teachers, assessed adaptive functioning in school
Tinelli et al. (2023)	RCT	Neurodevelopmental conditions, including autism and ADHD (Social communication) Early years, 2 years to 4 years and 11 months	Outpatient	Direct treatment sessions/home training programme	Parents	Usual care	Cost‐consequence analysis	Limited societal	6 months	Severity of autism symptoms: Assessed using the total score of social communication algorithm items from the Autism Diagnostic Observation Schedule‐Generic Child Language Parent–Child Dyadic Communication Social Difficulties Comorbid Psychopathology
Gibbard et al. (2004)	Non‐randomized controlled trial	Expressive language delay Early years, 22 to 36 months	Outpatient	Parent‐based intervention (PBI) consisting of 11 fortnightly group sessions with set language objectives for parents to work on at home with their child	Parents	Usual care	Cost‐Effectiveness Analysis	Healthcare system and families	8 months	Estimate of Phrase Length Word list Reynell Developmental Language Scales Pre‐School Language Scale—3 UK (comprehension and expression Mean length of utterance
Law et al. (2006)	Non‐randomized controlled trial	Primary language difficulties Early years	Early years setting, e.g., nurseries	Structured language curriculum with individualized planning, and daily intervention	Children	Usual care (Children receiving ‘typical’ provision in health service settings)	Cost consequence analysis*	Healthcare sector and families	6 months	Language and Behaviour Improvements
Frizelle et al. (2022)	Non‐randomized controlled trial	Universal intervention for children from disadvantaged areas Early years	Preschools	Community‐based language intervention with group training and individual coaching [“Happy Talk”]	Children and caregivers	Usual care	Cost‐utility analysis	Healthcare sector	8 months	Receptive language improvement: Pre‐school Language Scale 5th edition comprehension score Total language improvement: Pre‐school Language Scale 5th edition total score Health‐related quality of life: Child Health Utility instrument
Cronin and Addo (2021)	Longitudinal data‐ retrospective cohort study	Speech, language and communication needs Early years and school, for the 4 to 17 years	Other	Speech and language therapy	Not specified	N/A	Costing study	Justice system	Not specified	Youth antisocial behaviour: The study measured various antisocial behaviours in young people, including physical fights, skipping school, stealing, graffiti, carrying weapons, and more. (questionnaire) Youth Justice (YJ) Contacts: The study also examined contact with the system, including attending YJ conferences, being charged with offenses, appearing in court, and being convicted
Barnett et al. (1988)	RCT	Language handicaps (Language/communication difficulty (various definitions)) Early years, 35–59 months	Outpatient	(1) Home based (parent‐delivered) intervention, (2) centre‐based intervention, (3) both centre‐ and home‐based intervention	Parent	(4) No treatment (no intervention services from the clinic during the 13‐week period)	Cost consequence analysis*	Healthcare sector and patients/families	13 months	Auditory comprehension and verbal ability: Measured using the Preschool Language Scale—Revised Articulation proficiency: Assessed using the Arizona Articulation Proficiency Scale
Wake et al. (2015) link paper: Wake et al. (2012)	RCT	Language delay Early years, 4 to 6 years	Community	Home based therapy sessions × 18 one hour (language (narrative skills, vocabulary and grammar) and preliteracy skills (phonological awareness and letter knowledge) were targeted)	Children and caregivers	Usual care	Cost consequence analysis*	Healthcare system t and families	24 months	Expressive and receptive language: The trial evaluated both expressive and receptive language skills (CELF‐P2) Literacy skills: The study looked at literacy‐related skills, including word reading and spelling (Wide Range Achievement Test) Narrative skills: Researchers assessed narrative abilities, such as storytelling and understanding story structure (The Renfrew Language Scales: Bus Story Test) Phonological skills: Phonological awareness, which includes recognizing and manipulating sounds in words, was another outcome (Comprehensive Test of Phonological Processing) Pragmatic skills (social language use): The trial examined pragmatic language skills, which involve using language appropriately in social contexts (Children's Communication Checklist) Phonological short‐term memory (Children's Test of Non‐Word Repetition) Health‐Related Quality of Life: Parent‐reported measures assessed children's overall well‐being and quality of life (Health Utilities Index and Paediatric Quality of Life Inventory) Behaviour: The study considered behavioural aspects related to language delay and intervention effects (Strengths and Difficulties Questionnaire)
Popova et al. (2014)	Costing study	Other congenital disorders, including foetal alcohol syndrome and cerebral palsy Early years and school, 2 to 19 years	Other	1:1 speech‐language interventions (20–30 h of intervention depending on the severity)	Children	N/A	Costing study	Healthcare sector	12 months	Prevalence of Speech and Language Disorders (SLD) among Children with Foetal Alcohol Spectrum Disorder Severity Levels of Speech and Language disorders: The study categorized Speech and Language disorders severity into three levels: normal, mildly impaired, and moderately‐to‐severely impaired
Eiserman et al. (1992) Link paper: Eiserman, Weber, and McCoun (1995)	RCT	Moderate speech sound disorder Early years 3 to 4 years old	Community	Educational training mothers in therapeutic techniques (mom and child home training at least four times a week with 40‐min visits twice a month by a speech and language pathologist)	Parent	Standard Weekly speech and language therapy (occasional homework)	Cost consequence analysis*	Healthcare system and families	7 months	Battelle Developmental Inventory Goldman‐Fristoe Test of Articulation Patterned Syntax Elicitation Test Preschool Language Scale Test for Auditory Comprehension of Language Parenting Stress Index Family Adaptability and Cohesion Scales Structured Photographic Expressive Language Family Resource Scale Family Support Scale Family Inventory of Life Events
Actrn (2020)	Protocol: RCT	Other	Community	Online education online for parents with children (12–36 months) with communication difficulties [telehealth]	Parent	Face‐to‐face group workshop	Cost‐consequence analysis	Healthcare payer	N/R	Parent report of identification of actions in relation to parenting a child with communication difficulty, as assessed by 10 point Likert scale Attendance
Neumann (2022)	Protocol: RCT	Language disorder	Outpatient	Online interval small group of 2–3 children therapy (30 therapeutic sessions of 45 min each) [telehealth]	N/R	Standard face‐to‐face therapy (active intervention: 30 therapeutic sessions of 45 min each)	Cost‐effectiveness	Healthcare payer	N/R	Language development status Speech‐language therapists’ time consumption

*Note*: * Defined by the authors as a cost‐effectiveness study but did not actually combine cost and effectiveness outcomes. Grey area indicates a protocol for a study.

Abbreviations: QALYs: Quality‐Adjusted Life Years; RCT—Randomized controlled trial.

Studies varied in terms of SLT population of interest: language and/or communication difficulties (various definitions) (*n* = 6); speech sound disorder, including cleft speech (*n* = 2); paediatric dysphagia and/or feeding (*n* = 2); social communication in autism (*n* = 2); stammering (*n* = 1); foetal alcohol syndrome (*n* = 1); youth justice (*n* = 1); galactosemia (*n* = 1); universal services in an area of low socio‐economic status (*n* = 1). They also varied in terms of the interventions considered: various/unspecified (*n* = 13); Pre‐school Autism Communication Trial (PACT) (*n* = 2); and language intervention programme (*n* = 2).

Some common characteristics can nonetheless be identified, with most studies targeting language and/or communication (*n* = 12), followed by speech (*n* = 3), and swallowing /feeding (*n* = 2). Most were carried out with early years populations (*n* = 10) or early years and school‐aged children (*n* = 5), with one study focused on school‐aged children only, and one unspecified. In terms of the agent of intervention, many studies focused on indirect delivery (Parent and/or professional *n* = 10), whilst others on direct intervention (*n* = 7 SLT/early years language centre services).

The majority of studies either undertook a cost consequence analysis (*n* = 7) or costing study (*n* = 5). The remaining studies undertook a cost‐effectiveness analysis (*n* = 3) and cost‐utility analysis (*n* = 2). These cost evaluations were attached to a range of study designs, these were RCTs (*n* = 7), non‐randomized controlled trials (*n* = 3), before and after studies (*n* = 3), feasibility study (*n* = 1) and retrospective cohort study (*n* = 1). Two studies did not carry out any type of assessment of effectiveness. Most studies adopted a healthcare sector and patients/families (*n* = 6), limited societal perspective (*n* = 3), followed by the healthcare sector (*n* = 3), societal perspective (*n* = 2), teaching setting (*n* = 1), and the justice system (*n* = 1), and one study's specific perspective was difficult to identify. The time horizon of these studies ranged from 3 months to 24 months.

In addition to these studies, two RCTs’ protocols were registered and plan to examine the cost or cost‐effectiveness of telehealth compared to usual care. One of these studies will carry out a cost‐consequence analysis, and the other will be based on cost‐effectiveness. Both economic evaluations will take a healthcare payer perspective.

## 4 Discussion

This scoping review of economic evaluations of SLT interventions was initiated in response to the escalating pressures on health and social care services to demonstrate interventions that are not only effective but also represent value for money (Care Quality Commission 2017; Office for Health Improvement and Disparities 2020; The Kings Fund 2015). This reflects a broader demand for accountability and value in the provision of health and social care, including SLT. Regarding the SLT economic evidence base, economic research has now been undertaken for more than three decades. Over this period, a total of 44 completed studies and eight ongoing studies were identified assessing the cost or cost‐effectiveness of SLT interventions. Contextualizing the number of studies is challenging because scoping reviews typically differ significantly in the populations, interventions, and outcomes assessed (Tricco et al. 2018). However, previous scoping reviews of economic evaluations focusing on a single approach or intervention in other professions have identified a greater number of studies than our findings (Kasztura et al. 2019; Richardson et al. 2022; Xi et al. 2023). Given the range of clinical areas in which speech and language therapists work and various interventions utilized, a more substantial body of evidence would be expected from such a broad scoping review. However, recent trends indicate a growing recognition of the importance of undertaking economic evaluations. Furthermore, more recent studies assessing SLT interventions have utilized cost‐effectiveness and cost‐utility analyses.

Most of the included economic evaluations were undertaken in three main geographical regions, specifically Australia, the United States and the United Kingdom. Such a geographic focus has limitations. Although there are methods to convert regional costs and healthcare system parameters (e.g., purchasing power parity, inflation and price indices and regression‐based adjustment models), it is beneficial to have data that is specific to the jurisdiction in which the evaluation is being conducted, as different healthcare systems may not be comparable (Reinhold et al. 2010). As such, it is important to encourage economic evaluations of SLT interventions in different regions, especially where evidence is currently scarce but also more generally across the world. Despite the recent growth in economic evaluations in SLT, there is a lack of studies citing each other. The lack of interconnectivity may reflect the limited evidence base and the breadth of areas in which speech and language therapists work. This diversity spans multiple services (e.g., early years programs, prison services, social care settings, hospitals), targeting different concerns (e.g., speech, language, communication, swallowing) and employing various interventions (e.g., parent training, video fluoroscopic assessments, cognitive behavioural therapy, transcranial stimulation). The limited amount of interconnectivity along with the heterogeneity of populations and interventions may also indicate a need for greater coordination and collaboration.

Studies assessed the cost or cost‐effectiveness of SLT interventions for both adults and children. The focus in adult populations was predominantly on SLT interventions for stroke and, to a lesser extent, on head and neck cancer and Parkinson's disease. Despite this apparent consistency within the populations of interest, few studies explored the same interventions. A similar landscape of considerable heterogeneity was observed in SLT interventions for children, further complicated by significant variations in the populations of interest. Although some commonality was observed in the use of indirect delivery (SLT interventions through the training of a family member or carer), the specific interventions and corresponding mediating factors varied substantially.

Often, interventions are described only in terms of the level of service, such as the duration or quantity of SLT provided, rather than the specific nature of the intervention itself. This lack of specificity significantly hampers the repeatability of any study and pooling of data within a systematic review of economic evaluations. Moreover, the considerable variation in the techniques and methods of delivering interventions can lead to substantial heterogeneity in their effects. Therefore, there is limited opportunity for conducting a cost‐effectiveness assessment of a particular SLT intervention (Mandrik et al. 2021). However, there appears to be some consistent examination of the cost‐effectiveness of telehealth compared to traditional face‐to‐face delivery which may warrant further exploration and possible economic evidence synthesis. Furthermore, previous reviews have highlighted that the use of telehealth is growing within SLT (Molini‐Avejonas et al. 2015), and it has great potential regarding reducing costs for patients and healthcare systems due to reduced travel costs (Shahouzaie and Gholamiyan Arefi 2024).

Despite many studies describing their economic evaluations as cost‐effectiveness analyses, most studies did not combine cost data with effectiveness data to generate an incremental cost‐effectiveness ratio. Combining the effects and costs enhances comparison regarding the level of impact achieved relative to the investment in the intervention (Moayyedi and Mason 2004). Cost‐utility studies have the advantage of allowing direct comparison of different interventions using a common outcome, such as quality‐adjusted life years (Feng et al. 2020), helping decision‐makers assess whether the investment in that intervention is justified compared to investing in other alternatives (National Institute for Health and Care Excellence 2022a). Although cost‐effectiveness and cost‐utility studies have been relatively rare, it is evident that there has been an increase in recent years, indicating promising advancements in this area.

When undertaking these economic evaluations, the main perspectives used are either healthcare sector or payer perspectives. While a limited societal perspective focusing on individual costs incurred in receiving the intervention (e.g., travel) was adopted in some cases, a broader societal view that captures the wider ramifications of SLT benefits is often lacking. In many instances, this may reflect the requirements of the funder of the research or body providing guidance around the use of the interventions, as well as the added complexities of taking a societal perspective. Similarly, the time horizon chosen for the economic evaluations is often limited, with most studies restricted to the intervention period or a short follow‐up. Long‐term evaluations are crucial, as the effectiveness and cost savings, particularly from a societal perspective, may only be demonstrated over extended periods. There are studies identified in this review which consider a broader societal view and longer time horizon (e.g., Cronin and Addo 2021; Palmer et al. 2019), but this type of study is the exception. It is promising these economic evaluations of SLT are typically run alongside RCTs or non‐randomized controlled trials.

### 4.1 Future Research

Given the various areas for potential development, a coordinated, system‐wide approach may be warranted to broaden and deepen our understanding of the economic value of SLT interventions. As part of the efforts to standardize and enhance reporting, adopting the Template for Intervention Description and Replication (TIDieR) is recommended to improve the standardization of intervention reporting and delivery (Hoffmann et al. 2014). Similarly, the adoption of standardized reporting for economic evaluations would be beneficial (Husereau et al. 2022), as this adoption may help in improving transparency and repeatability of future studies (Hoffmann et al. 2014).

Where key areas are identified, there would be a need for high‐quality RCTs to be undertaken with full economic cost‐utility and cost‐effectiveness analyses using an appropriate perspective (whether healthcare payer or societal) and lifetime horizon. Where RCTs are not feasible, it is advisable to focus resources and funding on the development of economic models based on existing standardized interventions’ effectiveness evidence from RCTs or systematic reviews. The challenges associated with using a lifetime horizon have been identified (O'Mahony et al. 2015; Simpson et al. 2009); however, use of modelling techniques such as Markov models and discrete‐event simulations would enable long‐term projections of costs and effectiveness without the need for long‐term follow‐up (Gye et al. 2024; Sato and Zouain 2010; Wang et al. 2023). These approaches have limitations (Carta and Conversano 2020; Standfield et al. 2014); however, they can identify gaps in the current evidence base and aid in formulating recommendations for future research.

For this research to be successfully undertaken, a workforce development program is required to promote the conduct, necessity, and interpretation of economic evaluations of SLT interventions (De Vito et al. 2009; Ramani et al. 2019). This workforce development should occur at all levels of the profession, starting with basic knowledge during pre‐registration training, with priority and workforce planning facilitating more advanced knowledge for service leads and senior researchers. Collaborations with health economists will form a fundamental part of such a programme.

### 4.2 Strengths and Limitations

This scoping review possesses several methodological strengths. Initially, a protocol was preregistered before the review commenced. Furthermore, the review was conducted and reported following standardized methods (Levac et al. 2010; Peters et al. 2015; Tricco et al. 2018). A comprehensive multidata‐based two‐stage search strategy was undertaken, which included forward and backward citation screening (Higgins 2023). This search strategy was developed through a collaboration involving academic, clinical, and an information specialist. For the screening process, although only a single reviewer was used, a 10% verification process was undertaken, where substantial agreement was achieved among all reviewers. Unfortunately, due to restrictions in capacity, this review had some limitations. The search strategy only included English language studies, which may produce possible issues of language bias (Moher et al. 2003). The review represents the state of evidence as of the final search date. However, as time goes on, this review will become more out‐of‐date and may not represent the totality of all evidence within this field. Due to the substantial number of studies and the limited resources, the 10% verification process for data extraction was unable to be carried out. This could result in errors within the data items that were not double‐checked. However, all economic data items were reviewed by another reviewer (JH) and then again by an economic specialist (VB).

## 5 Conclusion

Whilst a small number of studies have started to evaluate economic aspects of SLT, there is a relative scarcity of evidence in this area, underscoring a need to prioritize further economic evaluation. Due to the substantial heterogeneity and lack of interconnectivity of the current evidence base, it is vital that this call to action is strategically organized to optimize and coordinate the work. Components of such a programme would include priority setting, national and international coordination, influencing and the establishment of key collaborations, networking, and workforce development. Economic evaluations should ideally run alongside RCTs, designed around cost‐effectiveness or cost‐utility analyses, and adopt a wider perspective and lifetime horizon through the use of decision‐analytic modelling techniques wherever feasible. In cases where RCTs are not possible, alternative economic modelling of interventions should be considered in relation to existing RCTs in the field.

## Conflicts of Interest

Amit Kulkarni and Gemma Jones are employed by the Royal College of Speech & Language Therapists.

## Supporting information



Supporting Information

## Data Availability

The data that support the findings of this systematic review are available on request from James Hill (Email: Jehill1@uclan.ac.uk).
