# Supplementary material for: Economic Evaluations of Speech and Language Therapy Interventions: A Scoping Review
Source: Int J Lang Commun Disord. 2025 Jul 12;60(4):e70091. doi: 10.1111/1460-6984.70091 (PMC12254717; doi:10.1111/1460-6984.70091)
Supplement: Supplementary file 1 — Supporting Information [file JLCD-60-0-s001.docx]

# Appendix 1: Data extraction form

| **Author** | **Year** | **DOI** | **Title** | **Link (Trial protocols)** | **Reviewer 1 initial** | Include (Full paper screening) | Nots | Aims of the study | Protocol only | Abstract Only | Country of study | Town/city Code | Town/city | Study type | Study type Code | Cost analysis method | Clinical setting | Clinical setting Code | Clinical setting Notes |
| --- | --- | --- | --- | --- | --- | --- | --- | --- | --- | --- | --- | --- | --- | --- | --- | --- | --- | --- | --- |
|  |  |  |  |  |  |  |  |  |  |  |  |  |  |  |  |  |  |  |  |

| Population (Patient/client where applicable) | Population (adult/children/mixed/other) | Population (Descriptor) | Nots | Full description of Intervention | Intervention type | Intervention type Code | Comparator (if applicable) | Mode of delivery | Individual being supported if applicable | Descriptive term for Speech and Language Therapist | perspective | Time horizon | Valuation of resources, costs and outcomes | Method of analysis | Outcomes | Measurement |
| --- | --- | --- | --- | --- | --- | --- | --- | --- | --- | --- | --- | --- | --- | --- | --- | --- |
|  |  |  |  |  |  |  |  |  |  |  |  |  |  |  |  |  |

# Appendix 2: Preferred Reporting Items for Systematic reviews and Meta-Analyses extension for Scoping Reviews (PRISMA-ScR) Checklist

| **SECTION** | **ITEM** | **PRISMA-ScR CHECKLIST ITEM** | **REPORTED ON PAGE #** |
| --- | --- | --- | --- |
| **TITLE** | | | |
| Title | 1 | Identify the report as a scoping review. | 1 |
| **ABSTRACT** | | | |
| Structured summary | 2 | Provide a structured summary that includes (as applicable): background, objectives, eligibility criteria, sources of evidence, charting methods, results, and conclusions that relate to the review questions and objectives. | 3 |
| **INTRODUCTION** | | | |
| Rationale | 3 | Describe the rationale for the review in the context of what is already known. Explain why the review questions/objectives lend themselves to a scoping review approach. | 4-5 |
| Objectives | 4 | Provide an explicit statement of the questions and objectives being addressed with reference to their key elements (e.g., population or participants, concepts, and context) or other relevant key elements used to conceptualize the review questions and/or objectives. | 5 |
| **METHODS** | | | |
| Protocol and registration | 5 | Indicate whether a review protocol exists; state if and where it can be accessed (e.g., a Web address); and if available, provide registration information, including the registration number. | 6 |
| Eligibility criteria | 6 | Specify characteristics of the sources of evidence used as eligibility criteria (e.g., years considered, language, and publication status), and provide a rationale. | 6-7 |
| Information sources* | 7 | Describe all information sources in the search (e.g., databases with dates of coverage and contact with authors to identify additional sources), as well as the date the most recent search was executed. | 6 |
| Search | 8 | Present the full electronic search strategy for at least 1 database, including any limits used, such that it could be repeated. | Supplement file 1 |
| Selection of sources of evidence† | 9 | State the process for selecting sources of evidence (i.e., screening and eligibility) included in the scoping review. | 7-8 |
| Data charting process‡ | 10 | Describe the methods of charting data from the included sources of evidence (e.g., calibrated forms or forms that have been tested by the team before their use, and whether data charting was done independently or in duplicate) and any processes for obtaining and confirming data from investigators. | 7 |
| Data items | 11 | List and define all variables for which data were sought and any assumptions and simplifications made. | 7-8 |
| Critical appraisal of individual sources of evidence§ | 12 | If done, provide a rationale for conducting a critical appraisal of included sources of evidence; describe the methods used and how this information was used in any data synthesis (if appropriate). | N/A |
| Synthesis of results | 13 | Describe the methods of handling and summarizing the data that were charted. | 8 |
| **RESULTS** | | | |
| Selection of sources of evidence | 14 | Give numbers of sources of evidence screened, assessed for eligibility, and included in the review, with reasons for exclusions at each stage, ideally using a flow diagram. | 9 - 10 |
| Characteristics of sources of evidence | 15 | For each source of evidence, present characteristics for which data were charted and provide the citations. | 14 - 70 |
| Critical appraisal within sources of evidence | 16 | If done, present data on critical appraisal of included sources of evidence (see item 12). | N/A |
| Results of individual sources of evidence | 17 | For each included source of evidence, present the relevant data that were charted that relate to the review questions and objectives. | 14 - 70 |
| Synthesis of results | 18 | Summarize and/or present the charting results as they relate to the review questions and objectives. | 14 - 70 |
| **DISCUSSION** | | | |
| Summary of evidence | 19 | Summarize the main results (including an overview of concepts, themes, and types of evidence available), link to the review questions and objectives, and consider the relevance to key groups. | 71 |
| Limitations | 20 | Discuss the limitations of the scoping review process. | 73 |
| Conclusions | 21 | Provide a general interpretation of the results with respect to the review questions and objectives, as well as potential implications and/or next steps. | 72 -74 |
| **FUNDING** | | | |
| Funding | 22 | Describe sources of funding for the included sources of evidence, as well as sources of funding for the scoping review. Describe the role of the funders of the scoping review. | 2 |

JBI = Joanna Briggs Institute; PRISMA-ScR = Preferred Reporting Items for Systematic reviews and Meta-Analyses extension for Scoping Reviews.

* Where *sources of evidence* (see second footnote) are compiled from, such as bibliographic databases, social media platforms, and Web sites.

† A more inclusive/heterogeneous term used to account for the different types of evidence or data sources (e.g., quantitative and/or qualitative research, expert opinion, and policy documents) that may be eligible in a scoping review as opposed to only studies. This is not to be confused with *information sources* (see first footnote).

‡ The frameworks by Arksey and O’Malley (6) and Levac and colleagues (7) and the JBI guidance (4, 5) refer to the process of data extraction in a scoping review as data charting*.*

§ The process of systematically examining research evidence to assess its validity, results, and relevance before using it to inform a decision. This term is used for items 12 and 19 instead of "risk of bias" (which is more applicable to systematic reviews of interventions) to include and acknowledge the various sources of evidence that may be used in a scoping review (e.g., quantitative and/or qualitative research, expert opinion, and policy document).

*From:* Tricco AC, Lillie E, Zarin W, O'Brien KK, Colquhoun H, Levac D, et al. PRISMA Extension for Scoping Reviews (PRISMAScR): Checklist and Explanation. Ann Intern Med. 2018;169:467–473. [doi: 10.7326/M18-0850](http://annals.org/aim/fullarticle/2700389/prisma-extension-scoping-reviews-prisma-scr-checklist-explanation).

# Appendix 2: Ovid MEDLINE Search Strategy

Ovid MEDLINE® ALL <1946 to October 19, 2023>

1 Speech Therapy/

2 Language Therapy/

3 ((speech or language or voice) adj3 (therap* or intervention* or treatment* or patholog* or rehabilitation or train* or clinic or clinics or service* or centre*1 or center*1)).ti,ab,kf.

4 (speech therap* or language therap*).in.

5 or/1-4

6 Economics/

7 exp “Costs and Cost Analysis”/

8 Economics, Nursing/

9 Economics, Medical/

10 Economics, Pharmaceutical/

11 exp Economics, Hospital/

12 Economics, Dental/

13 exp “Fees and Charges”/

14 exp Budgets/

15 budget*.ti,ab,kf.

16 (economic* or cost or costs or costly or costing or price or prices or pricing or pharmacoeconomic* or pharmaco-economic* or expenditure or expenditures or expense or expenses or financial or finance or finances or financed).ti,kf.

17 (economic* or cost or costs or costly or costing or price or prices or pricing or pharmacoeconomic* or pharmaco-economic* or expenditure or expenditures or expense or expenses or financial or finance or finances or financed).ab. /freq=2

18 (cost* adj2 (effective* or utilit* or benefit* or minimi* or analy* or outcome or outcomes)).ab,kf.

19 (value adj2 (money or monetary)).ti,ab,kf.

20 exp models, economic/

21 economic model*.ab,kf.

22 markov chains/

23 markov.ti,ab,kf.

24 monte carlo method/

25 monte carlo.ti,ab,kf.

26 exp Decision Theory/

27 (decision* adj2 (tree* or analy* or model*)).ti,ab,kf.

28 or/6-27

29 5 and 28

30 limit 29 to english language

# Appendix 3: Reasons for exclusion at full paper screening

| Author | Year | DOI | Title | Link (Trial protocols) | Reviewer 1 initial | Include (Full paper screening) | Nots |
| --- | --- | --- | --- | --- | --- | --- | --- |
| Actrn | 2015 | doi: | COMPARE - An evaluation of two different treatments compared to usual speech pathology in people with problems communicating after stroke | <https://www.cochranelibrary.com/web/cochrane/content?templateType=full&urlTitle=%2Fcentral%2Fdoi%2F10.1002%2Fcentral%2FCN-02442006&doi=10.1002%2Fcentral%2FCN-02442006&type=central&contentLanguage=> | JH | No | Study type |
| Ali, M., Ben Basat, A. Lifshitz, Berthier, M., Blom Johansson, M., Breitenstein, C., Cadilhac, D. A., Constantinidou, F., Cruice, M., Davila, G., Gandolfi, M., Gil, M., Grima, R., Godecke, E., Jesus, L., Jiminez, L. Martinez, Kambanaros, M., Kukkonen, T., Laska, A., Mavis, I., Mc Menamin, R., Mendez-Orellana, C., Obrig, H., Ostberg, P., Robson, H., Sage, K., Van De Sandt-Koenderman, M., Sprecht, K., Visch-Brink, E., Wehling, E., Wielaert, S., Wallace, S. J., Williams, L. J. and Brady, M. C. | 2021 | 10.1080/02687038.2021.1914813 | Protocol for the development of the international population registry for aphasia after stroke (I-PRAISE) |  | JH | No | Study type |
| Anonymous | 2022 | 10.1055/s-0042-1748191 | Health Economics in Speech-Language Pathology |  | LS | No | Study type |
| Barrett, E. E. and ur, D. | 2014 | 10.1007/s00455-014-9571-4 | Dysphagia Research Society Annual Meeting and Post-Graduate Course |  | JH | No | Study type (indicates radiology cost) |
| Bidmead, Elaine, Nick Harrop, Dr, Reid, Tilly, Marshall, Alison and Southern, Veronica | 2015 | 10.1108/cgij-06-2015-0020 | “Teleswallowing”: a case study of remote swallowing assessment |  | LS | No | Study type |
| Born, H. L., De Alarcon, A., Tabangin, M., Zacharias, S. and Kelchner, L. | 2017 | 10.1177/0194599817717251 | Business of Medicine/Practice Management |  | LS | No | Incorrect intervention |
| Brogan, Emily L., Kim, Joosup, Grimley, Rohan S., Wallace, Sarah J., Baker, Caroline, Thayabaranathan, Tharshanah, Andrew, Nadine E., Kilkenny, Monique F., Godecke, Erin, Rose, Miranda L. and Cadilhac, Dominique A. | 2023 |  | The Excess Costs of Hospitalization for Acute Stroke in People With Communication Impairment: A Stroke123 Data Linkage Substudy | | JH | no | intervention |
| Collins, A., Burns, C. L., Ward, E. C., Comans, T., Blake, C., Kenny, L., Greenup, P. and Best, D. | 2017 | 10.1177/1357633X17733020 | Home-based telehealth service for swallowing and nutrition management following head and neck cancer treatment |  | LS | No | Intervention: |
| Cronin, P., Reeve, R., McCabe, P., Viney, R. and Goodall, S. | 2017 |  | The impact of childhood language difficulties on healthcare costs from 4 to 13 years: Australian longitudinal study | | JH | no | intervention |
| Crook, C. L. and Simpson, B. | 2011 | 10.1111/j.1747-4949.2011.00684.x | UK Stroke Forum 2011 Abstracts, Glasgow, 29 November 2011–1 December 2011 |  | LS | No | Study type |
| Cup, E. H., Pieterse, A. J., Knuijt, S., Hendricks, H. T., van Engelen, B. G., Oostendorp, R. A. and van der Wilt, G. J. | 2007 | 10.1080/09638280600926702 | Referral of patients with neuromuscular disease to occupational therapy, physical therapy and speech therapy: usual practice versus multidisciplinary advice |  | LS | No | Intervention: |
| Dowling, F., Clarke, C. E., Patel, S., Woolley, R., Ives, N. J., Rick, C. E., Wheatley, K., Smith, C., Brady, M. and Sackley, C. M. | 2014 | 10.1002/mds.25914 | Abstracts of the Eighteenth International Congress of Parkinson's Disease and Movement Disorders, June 8-12, 2014, Stockholm, Sweden |  | JH | No | Study type |
| Ellis, C., Hardy, R. Y. and Lindrooth, R. C. | 2017 |  | Greater healthcare utilization and costs among Black persons compared to White persons with aphasia in the North Carolina stroke belt |  | JH | no | intervention |
| Ellis, C., Lindrooth, R. C. and Horner, J. | 2014 | 10.1044/2013_AJSLP-13-0037 | Retrospective cost-effectiveness analysis of treatments for aphasia: an approach using experimental data |  | JH | No | Study type |
| Ellis, C., Simpson, A. N., Bonilha, H., Mauldin, P. D. and Simpson, K. N. | 2012 |  | The One-Year Attributable Cost of Poststroke Aphasia |  | JH | no | intervention |
| Ellis, Charles, Peach, Richard K., Hardy, Rose Y. and Lindrooth, Richard C. | 2017 link | 10.1080/02687038.2017.1303440 | The influence of race on SLP utilisation and costs among persons with aphasia |  | JH | No | Study type |
| Garcia, Jane Mertz, Garrett, Kathryn L., Pimentel, Jane T. and Garcia, Robert G. | 2002 | 10.1080/02687030143000852 | Clinical services for aphasia: A survey of university clinics |  | VB | No | Study type |
| Gaustad, J. V., Kleven, L., Kornør, H., Harboe, I., Flatby, A. V., Aakhus, E., Bystad, M. and Røste, I. | 2022 | doi: | Transkraniell likestrømsbehandling for depresjon og afasi: fullstendig metodevurdering |  | VB | No | intervention |
| Jacobs, Molly and Ellis, Charles | 2023 |  | Aphasianomics: estimating the economic burden of poststroke aphasia in the United States | | JH | no | intervention |
| Konst, Emmy M., Prahl, Charlotte, Weersink-Braks, Hanny, De Boo, Theo, Prahl-Andersen, Birte, Kuijpers-Jagtman, Anne M. and Severens, Johan L. | 2004 | doi: | Cost-effectiveness of infant orthopedic treatment regarding speech in patients with complete unilateral cleft lip and palate: a randomized three-center trial in the Netherlands (Dutchcleft) |  | AK | No | intervention |
| Law, J., Zeng, B., Lindsay, G. and Beecham, J. | 2012 | 10.1111/j.1460-6984.2011.00084.x | Cost-effectiveness of interventions for children with speech, language and communication needs (SLCN): a review using the Drummond and Jefferson (1996) 'Referee's Checklist' |  | AK | No | Study type |
| Mohammadpour, F., Basakha, M., Kamal, S. H. M. and Azari, N. | 2022 | 10.1186/s12962-022-00366-z | Costing the outpatient rehabilitation services: time-driven activity-based costing approach |  | DM | No | intervention |
| Moroney, L. B., Ward, E. C., Helios, J., Crombie, J., Burns, C. L., Blake, C., Comans, T., Chua, B., Kenny, L. and Hughes, B. G. M. | 2020 | 10.1007/s00520-019-04992-x | Evaluation of a speech pathology service delivery model for patients at low dysphagia risk during radiotherapy for HNC |  | DM | No | intervention |
| Murray, S., Ginnelly, A. and Patel, K. | 2019 | 10.1016/j.physio.2018.11.194 | A therapy assistant in the intensive care unit (ICU): a pilot project |  | DM | No | intervention |
| n9y, R. B. R. | 2023 | doi: | Effect of Transcranial Stimulation associated with Speech Therapy on swallowing in the acute phase of Stroke | <https://ensaiosclinicos.gov.br/rg/RBR-72n9y83> | DM | No | intervention |
| Nct | 2002 | doi: | A Comparison of Language Intervention Programs | <https://www.cochranelibrary.com/central/doi/10.1002/central/CN-01508846/full> | DM | No | intervention |
| Nct | 2017 | doi: | Adapted Solution Focused Therapy for People With Aphasia (SOFIA Trial) | <https://www.cochranelibrary.com/central/doi/10.1002/central/CN-01495612/full> | DM | No | Study type |
| Nct | 2020 | doi: | Telerehabilitation for Post-stroke Patients | [https://www.cochranelibrary.com/central/doi/10.1002/central/CN-02125127/full](https://eur01.safelinks.protection.outlook.com/?url=https%3A%2F%2Fwww.cochranelibrary.com%2Fcentral%2Fdoi%2F10.1002%2Fcentral%2FCN-02125127%2Ffull&data=05%7C01%7CCHarris10%40uclan.ac.uk%7C6c9c2a0cf2bf41ae507c08dbef621e81%7Cebf69982036b4cc4b2027aeb194c5065%7C0%7C0%7C638366976919752464%7CUnknown%7CTWFpbGZsb3d8eyJWIjoiMC4wLjAwMDAiLCJQIjoiV2luMzIiLCJBTiI6Ik1haWwiLCJXVCI6Mn0%3D%7C3000%7C%7C%7C&sdata=qcHAUDOKqVWmlkl8EUQ9qu9JITq0QwcM9V02Wv1GUjA%3D&reserved=0) | DM | No | intervention |
| Nct | 2022 | doi: | Parent-implemented Social Communication Treatment in Preschool Children With Autism Spectrum Disorder | <https://www.cochranelibrary.com/central/doi/10.1002/central/CN-02504108/full> | DM | No | Study type |
| Papathanasiou, I. and Heron, C. | 1998 | 10.3109/13682829809179418 | Case weighting in a speech and language therapy service |  | LM | No | Study type |
| Pennington, L., Roddam, H., Burton, C., Russell, I., Godfrey, C. and Russell, D. | 2005 | 10.1191/0269215505cr878oa | Promoting research use in speech and language therapy: a cluster randomized controlled trial to compare the clinical effectiveness and costs of two training strategies |  | LM | No | Study type |
| Schwarz, M., Coccetti, A., Cardell, E., Murdoch, A. and Davis, J. | 2017 | 10.1080/17549507.2016.1221457 | Management of swallowing in thrombolysed stroke patients: Implementation of a new protocol |  | GC | No | Study type |
| Sitzman, T. and Britto, M. | 2016 | doi: | Cost effectiveness of alternative strategies in the treatment of velopharyngeal insufficiency following cleft palate repair |  | GC | No | intervention |
| Slovarp, L. J., Jette, M. E., Gillespie, A. I., Reynolds, J. E. and Barkmeier-Kraemer, J. M. | 2021 | 10.1007/s00408-021-00442-w | Evaluation and Management Outcomes and Burdens in Patients with Refractory Chronic Cough Referred for Behavioral Cough Suppression Therapy |  | GC | No | Study type |
| Tam, A., Mac, S., Isaranuwatchai, W. and Bayley, M. | 2019 |  | Cost-effectiveness of a high-intensity rapid access outpatient stroke rehabilitation program | | JH | no | intervention |
| Tarrant, M., Carter, M., Dean, S. G., Taylor, R. S., Warren, F. C., Spencer, A., Adamson, J., Landa, P., Code, C. and Calitri, R. | 2018 | 10.1136/bmjopen-2018-025167 | Singing for people with aphasia (SPA): a protocol for a pilot randomised controlled trial of a group singing intervention to improve well-being |  | GC | No | intervention |
| Vallentin, T., Packham, T., Fleck, R. and Turkstra, L. | 2018 | 10.1177/1747493018789543 | World Stroke Congress Abstracts, 2018 |  | GJ | No | Study type |
| van der Gaag, A. and Brooks, R. | 2008 | 10.1080/13682820701560376 | Economic aspects of a therapy and support service for people with long-term stroke and aphasia |  | GJ | No | intervention |
| Vohr, B. R., Oh, W., Stewart, E. J., Bentkover, J. D., Gabbard, S., Lemons, J., Papile, L. A. and Pye, R. | 2001 |  | Comparison of costs and referral rates of 3 universal newborn hearing screening protocols | | JH | no | intervention |
| Wall, L. R., Kularatna, S., Ward, E. C., Cartmill, B., Hill, A. J., Isenring, E., Byrnes, J. and Porceddu, S. V. | 2019 | 10.1007/s00455-018-9960-1 | Economic Analysis of a Three-Arm RCT Exploring the Delivery of Intensive, Prophylactic Swallowing Therapy to Patients with Head and Neck Cancer During (Chemo)Radiotherapy |  | GJ | No | intervention |
| Weirather, Y. P., Korth, N., White, K. R., Downs, D. and WoodsKershner, N. | 1997 |  | Cost analysis of TEOAE-based universal newborn hearing screening | | JH | no | intervention |
| Weiss, Rita S. | 1980 | doi: | Efficacy and Cost Effectiveness of an Early Intervention Program for Young Handicapped Children |  | GJ | No (Could not retrieve) | cannot locate paper |
| Weiss, Rita S. | 1980 | doi: | Efficacy of INREAL Intervention for Preschool and Kindergarten Language Handicapped and Bilingual (Spanish) Children |  | GJ | No (Could not retrieve) | cannot locate paper |
| Werbaneth, K., Shum, J., Deane, S., Larrenaga, M., Tse, J. R., Bernier, E. and Vora, N. | 2018 | doi: | Reducing costs and length of stay using standardized dysphagia evaluation in acute stroke patients |  | GJ | No | Intervention |
| Yang, H. L., Yong, H. H. and Tan, D. | 2011 | doi: | Reducing nursing home pneumonias: A cost-effective approach |  | GJ | No | Intervention |
